# Cognitive Function and Variability in Antipsychotic Drug–Naive Patients With First-Episode Psychosis: A Systematic Review and Meta-Analysis

**DOI:** 10.1001/jamapsychiatry.2024.0016

**Published:** 2024-02-28

**Authors:** Maria Lee, Martin Cernvall, Jacqueline Borg, Pontus Plavén-Sigray, Cornelia Larsson, Sophie Erhardt, Carl M. Sellgren, Helena Fatouros-Bergman, Simon Cervenka

**Affiliations:** 1 Centre for Psychiatry Research, Department of Clinical Neuroscience, Karolinska Institutet, and Stockholm Health Care Services, Region Stockholm, Sweden; 2 Department of Medical sciences, Psychiatry, Uppsala University, Uppsala, Sweden; 3 Center for Cognitive and Computational Neuropsychiatry, Department of Clinical Neuroscience, Karolinska Institutet, & Stockholm Health Care Services, Region Stockholm, Sweden; 4 Neurobiology Research Unit, Copenhagen University Hospital, Rigshospitalet, Copenhagen, Denmark; 5 Department of Physiology and Pharmacology, Karolinska Institutet, Stockholm, Sweden

## Abstract

**Importance:**

Cognitive impairment contributes significantly to clinical outcome and level of function in individuals with psychotic disorders. These impairments are present already at psychosis onset at a group level; however, the question of heterogeneity in cognitive function among patients has not been systematically investigated.

**Objective:**

To provide an updated quantification of cognitive impairment at psychosis onset before patients receive potentially confounding antipsychotic treatment, and to investigate variability in cognitive function compared with healthy controls.

**Data Sources:**

In this systematic review and meta-analysis, PubMed articles were searched up to September 15, 2022.

**Study Selection:**

Original studies reporting data on cognitive function in antipsychotic drug–naive patients with first-episode psychosis (FEP) were included.

**Data Extraction and Synthesis:**

Data were independently extracted by 2 researchers. Cognitive tasks were clustered according to 6 domains of the Measurement and Treatment Research to Improve Cognition in Schizophrenia (MATRICS) Consensus Cognitive Battery and the domain of executive function. Random-effects model meta-analyses of mean differences and coefficient of variation ratios (CVRs) were performed, as well as meta-regressions, assessment of study quality, and publication bias.

**Main Outcomes and Measures:**

The main outcome measure was Hedges *g* for mean differences in cognition and CVR for within-group variability.

**Results:**

Fifty studies were included in the analysis with a total of 2625 individuals with FEP (mean [SD] age, 25.2 [3.6] years, 60% male; 40% female) and 2917 healthy controls (mean [SD] age, 26.0 [4.6]; 55% male; 45% female). In all cognitive domains, the FEP group displayed significant impairment compared with controls (speed of processing: Hedges *g* = −1.16; 95% CI, −1.35 to −0.98; verbal learning: Hedges *g* = −1.08; 95% CI, −1.28 to −0.88; visual learning: Hedges *g* = −1.05; 95% CI, −1.27 to −0.82; working memory: Hedges *g* = −1.04; 95% CI, −1.35 to −0.73; attention: Hedges *g* = −1.03; 95% CI, −1.24 to −0.82; reasoning/problem solving: Hedges *g* = −0.90; 95% CI, −1.12 to −0.68; executive function: Hedges *g* = −0.88; 95% CI, −1.07 to −0.69). Individuals with FEP also exhibited a larger variability across all domains (CVR range, 1.34-1.92).

**Conclusions and Relevance:**

Results of this systematic review and meta-analysis identified cognitive impairment in FEP before the initiation of antipsychotic treatment, with large effect sizes. The high variability within the FEP group suggests the need to identify those individuals with more severe cognitive problems who risk worse outcomes and could benefit the most from cognitive remediation.

## Key Points

**Question:**

How do patients with first-episode psychosis (FEP) compare with controls in cognitive function and cognitive variability, before initiation of antipsychotic medication?

**Findings:**

In this systematic review and meta-analysis including 50 studies and 2625 individuals with FEP, the mean cognitive performance of antipsychotic drug–naive individuals with FEP was significantly impaired compared with controls, across all cognitive domains. At the same time, the within-group variability in FEP was significantly greater, indicating a wider distribution of cognitive ability in patients with FEP compared with controls.

**Meaning:**

Patients with psychosis display cognitive difficulties very early in the disease process, and the considerable heterogeneity in cognitive function should prompt individual assessments in clinical settings in order to optimize care.

## Teaser

This systematic review and meta-analysis investigates cognitive function and variability before initiation of antipsychotic medication in patients with first-episode psychosis compared with controls.

## Introduction

Cognitive impairment is a central characteristic of schizophrenia^1,2,3^ and other psychotic disorders and has a strong association with functional^4,5,6^ and clinical outcome.^5,7^ Cognitive symptoms may often precede illness onset,^8,9,10^ and a previous meta-analysis^11^ showed significantly impaired cognitive performance already present in antipsychotic drug–naive patients with first-episode psychosis (FEP), ie, before the introduction of medication effects or adverse effects as potential confounders. In the study including reports published up until 2012, cognition was found to be impaired in patients, with medium to large effect sizes (standardized mean difference [SMD] range, −0.74 to −1.03). These results were comparable with earlier meta-analyses in FEP, which also included patients taking medication (eg, Mesholam-Gately and colleagues^12^ with an SMD range of −0.71 to −1.2), whereas a more recent study^13^ focusing only on Chinese samples assessed with the Measurement and Treatment Research to Improve Cognition in Schizophrenia (MATRICS) Consensus Cognitive Battery^14^(MCCB) produced slightly larger effect sizes (SMD range, −0.87 to −1.41). Importantly, all of these studies focused on mean comparisons only, despite the fact that psychotic disorders are generally considered to be highly heterogeneous in terms of symptom profile and course of illness.^15^

Attempts to describe variability in cognitive function have been synthesized in a systematic review of studies in schizophrenia spectrum and bipolar disorder^16^ and a narrative review on schizophrenia.^17^ Both reviews focused on studies investigating cognitive subgroups and concluded that distinct subgroups exist ranging from relative intact cognition to severe cognitive dysfunction. However, this body of research has typically included patients with longer duration of illness, and the question of heterogeneity early in the illness trajectory and before initiation of antipsychotic treatment is still unclear. Furthermore, due to the methodological variability in studies examining potential subgroups, meta-analytical evidence is still lacking regarding cognitive heterogeneity.

One way to measure heterogeneity is through comparing the amount of variability in the group of patients with psychosis with the variability in the control group, eg, by using the coefficient of variation ratio (CVR).^18^ This method was recently used by Catalan et al^19^ to demonstrate greater variability in cognitive function in individuals at clinical high risk for psychosis (CHR-P) compared with controls. Individuals at CHR-P are defined using different combinations of risk factors, and current estimates indicate that 25% go on to develop psychosis within a 3-year period.^20^ The study examining cognitive variability in those at CHR-P also included a small group of patients with FEP who exhibited similar within-group variability as those at CHR-P, but to our knowledge, cognitive variability in FEP has not been analyzed at the meta-analytic level in larger samples.

Since the previous meta-analysis of antipsychotic drug–naive individuals with FEP,^11^ a large number of new studies assessing cognitive deficits in patients have been published. The field has also seen an increased trend toward data harmonization since MCCB has become the dominant test battery. In addition, guidelines and standards for the reporting of systematic reviews have been updated.^21^ In the present study, our primary aim was, therefore, to update and extend the previous meta-analysis in antipsychotic drug–naive patients with FEP. The secondary aim was to perform the first systematic meta-analysis of within-group variability in cognitive ability in FEP.

## Methods

The meta-analysis was registered on PROSPERO and followed the Preferred Reporting Items for Systematic Reviews and Meta-analyses (PRISMA)^21^ (eTable 1 in Supplement 1) and Meta-Analysis of Observational Studies in Epidemiology (MOOSE)^22^ reporting guidelines (eTable 2 in Supplement 1).

### Search Strategy and Selection Criteria

We applied the same search terms as in our previous meta-analysis on antipsychotic drug–naive individuals with FEP^11^ (full search terms available in the eMethods 1 in Supplement 1). A literature search was performed on September 15, 2022, using the PubMed database. All abstracts were screened by 2 authors independently (M.L. and C.L.), using Rayyan software.^23^ Studies included in the meta-analysis fulfilled the following inclusion criteria: (1) available data on individuals with psychosis spectrum disorder (corresponding to any diagnosis within the *DSM-IV* chapter on “Psychotic Disorders” excluding a diagnosis of psychotic disorder due to a general medical condition and substance-induced psychotic disorder), (2) individuals categorized as having FEP, (3) patients reported as being antipsychotic-drug naive, (4) available data on cognitive performance, (5) available cognitive data from a healthy control group, and (6) ability to cluster cognitive tests into 1 of 6 neurocognitive categories of the MCCB or executive function. Exclusion criteria were (1) articles in a language other than English, (2) review articles or case reports, (3) overlapping sample and neurocognitive test, and (4) cohorts with a mean duration of untreated psychosis (DUP) longer than 5 years. Studies deemed relevant were read in full length. Reference lists were screened, and known researchers who had previously published on antipsychotic drug–naive samples were contacted. Conflicts regarding inclusion were resolved through discussion. For studies published before 2012, we relied on data reported in Fatouros-Bergman et al^11^ (eMethods 2 in Supplement 1). Race and ethnicity data were not collected in our study. Swedish records do not track race or ethnicity, and we did not consider it a primary aspect of our research question.

### Dataset From the Karolinska Schizophrenia Project

In addition to the literature search, we also included our own cognitive data collected within the Karolinska Schizophrenia Project (KaSP; antipsychotic drug–naive patients with FEP and age- and sex-matched controls), parts of which have been published elsewhere^24,25^ (eMethods 3 in Supplement 1).

### Outcome Measures and Data Extraction

Cognitive tests were grouped in a similar way as in Fatouros-Bergman et al^11^ (eMethods 2 in Supplement 1), following the domains of the MCCB, with the exception of social cognition. This was considered beyond the scope of this review, given the many different aspects (and outcome measures) of social cognition available.^26^ In addition, the domain of executive function was added. The executive function domain includes tests and outcome measures with a focus on cognitive flexibility, compared with the MCCB reasoning and problem-solving domain, which focuses more on organizational and planning skills. In summary, tests are grouped according to 7 domains: processing speed, attention, working memory, verbal learning, visual learning, reasoning and problem solving, and executive function. A complete list of outcome measures clustered under their respective domain is available in the eMethods 4 and eTable 9 in Supplement 1.

Two researchers (M.L. and M.C.) independently extracted data from the sample of studies into separate spreadsheets. Cognitive performance (mean and SD) was extracted for each eligible task, as well as demographic data (age, sex, years of education, DUP, country). The independent spreadsheets were then compared, and any inconsistencies were resolved through discussion.

When additional information was required, corresponding authors were contacted by email. If the FEP sample consisted of both antipsychotic drug–naive patients and patients either taking medication or drug free, data on only antipsychotic drug–naive individuals was requested. Raw scores or uncorrected T scores were requested when not already available. When several articles used patients from the same clinical institution, authors were asked to clarify whether samples were overlapping or not. If there was no response (after 1 additional reminder email), it was assumed that samples were overlapping (eTable 10 in Supplement 1). In all cases of overlapping samples and cognitive tests, the study with the largest sample size was chosen for inclusion in the meta-analysis.

### Statistical Analysis

Our main meta-analytical effect size measure was Hedges *g*. When applicable, test scores were transformed so that higher values always indicated better performance. Random-effects meta-analyses, using a restricted maximum likelihood random-effects variance estimator, were conducted for each of the 7 cognitive domains, resulting in a pooled estimate. For several of the domains (speed of processing, working memory, and executive function), participants could contribute with several outcome measures or test scores. To handle this unit of analysis problem,^27^ multilevel meta-analyses were conducted using the rmv.mv function in the metafor package, version 3.8-1^28^ in R (R Core Team), accounting for nonindependent effects and sampling errors (eMethods 5 in Supplement 1). Between-study heterogeneity was assessed using the *Q* statistic, the *I*^2^ index, and prediction intervals. The presence of publication bias was assessed by visually inspecting funnel plots. Study quality was judged using a modified version of the Newcastle-Ottawa Scale (eMethods 6 in Supplement 1). Two researchers (M.L. and M.C.) independently rated all studies and, when ratings differed, reached consensus through discussion. When 10 or more studies reported scores from the same cognitive test, meta-regressions were performed to evaluate the impact of a series of preregistered potential moderators. Age, sex, education, and year of publication were selected in order to replicate and extend the previous meta-analysis of cognitive function in antipsychotic drug–naive individuals with FEP by Fatouros-Bergman et al,^11^ and in addition, we included study quality (eMethods 8 in Supplement 1). To calculate and compare the within-group variability in FEP and controls, we used the CVR. The CVR is the natural logarithm of the ratio of estimates of population coefficients of variation,^18^ which is the ratio of the SDs normalized to the mean. Given the results of the prior meta-analysis,^11^ large differences in mean values were expected, and to get an unbiased estimate of variability, the CVR was deemed particularly appropriate in this case.

For robustness check, analyses of the log variability ratio were also conducted and can be found in eTable 13 in Supplement 1, along with CVR sensitivity analyses for domains containing negative outcome measures (eMethods 7 in Supplement 1). The CVR values presented in writing and plots have been back transformed from the log scale, to aid interpretability. All analyses were conducted using the metafor package^28^ in R, version 4.2.1 (R Core Team).^29^ Code for reproducing the analyses and figures can be found at Github.^30^

## Results

### Sample

**Figure 1.**
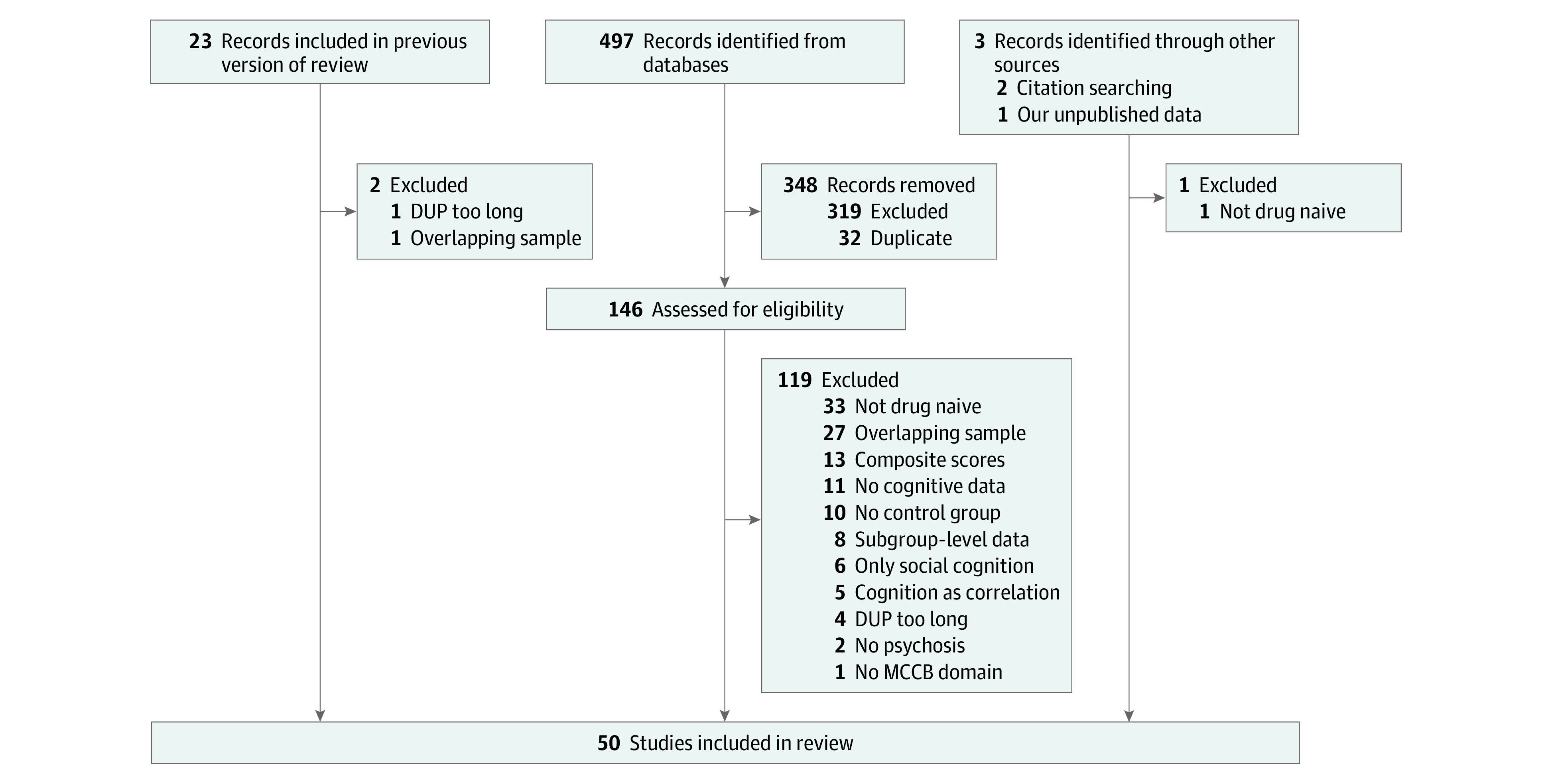
Adapted Version of the PRISMA 2020 Flow Diagram for Updated Systematic Reviews Including Searches of Databases, Registers, and Other Sources DUP indicates duration of untreated psychosis; MCCB, Measurement and Treatment Research to Improve Cognition in Schizophrenia Consensus Cognitive Battery.

In addition to the literature search, we included our own cognitive data collected within the Karolinska Schizophrenia Project (KaSP; 42 antipsychotic drug–naive patients with FEP and 64 age- and sex-matched controls) (eTables 3-8 in Supplement 1). The final sample consisted of 50 studies (Figure 1 and eTable 11 in Supplement 1), with a total of 2625 patients with FEP (mean [SD] age, 25.2 [3.6] years, 60% male; 40% female) and 2917 healthy controls (mean [SD] age, 26.0 [4.6]; 55% male; 45% female). The mean (SD) education level was 11.9 (1.3) years for patients and 13.2 (1.8) years for controls. Mean (SD) DUP was 20.9 (16.8) months (the number of studies available for analysis of DUP = 24).

### Cognitive Functioning in Antipsychotic Drug–Naive Patients With FEP as Compared With Controls

**Figure 2.**
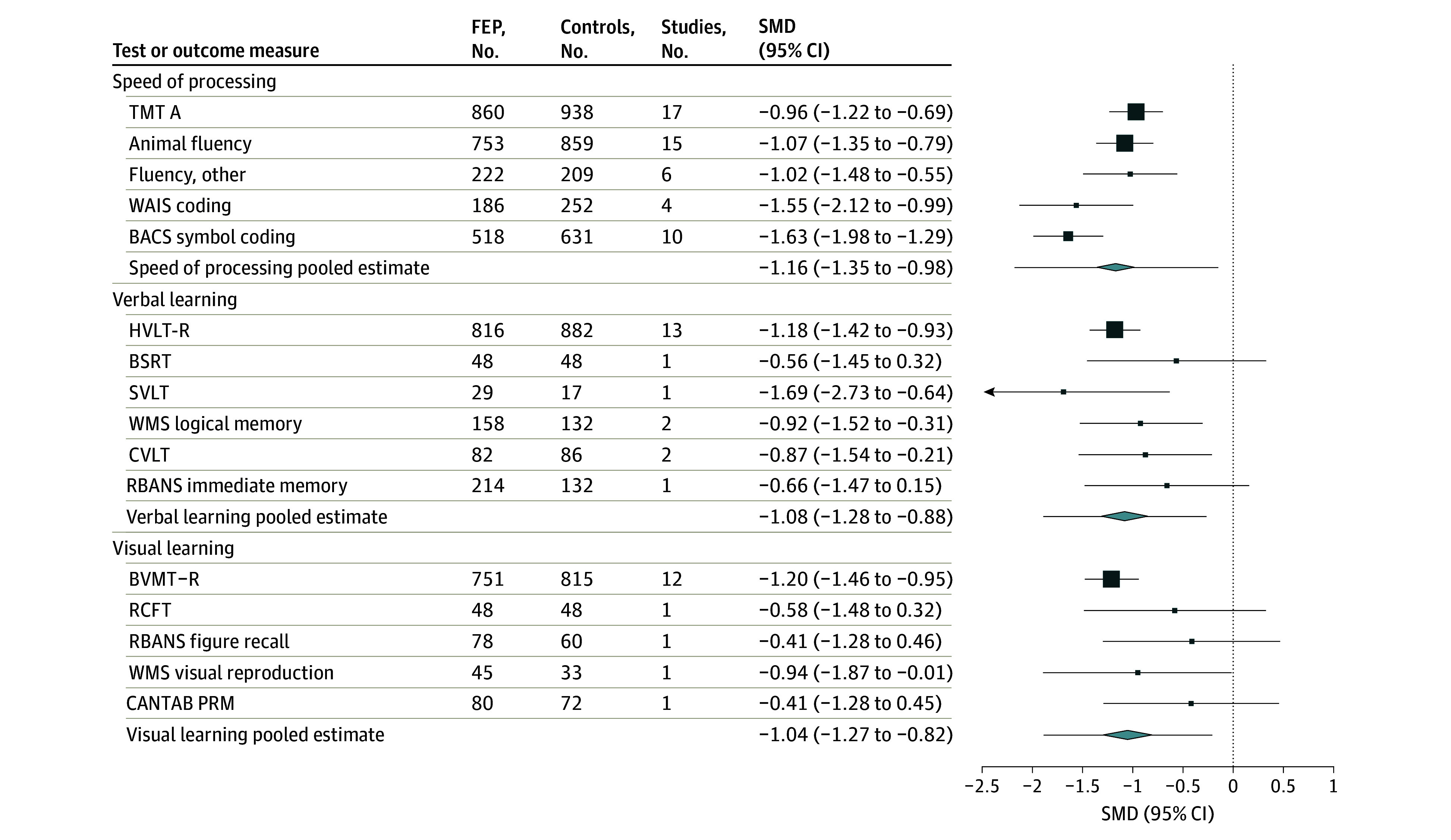
Combined Forest Plot of Mean Differences in Cognitive Ability for Speed of Processing, Verbal Learning, and Visual Learning Domains in Drug-Naive Patients With First-Episode Psychosis (FEP) Compared With Controls The lines extending from the pooled estimates indicate prediction interval. BACS indicates Brief Assessment of Cognition in Schizophrenia; BSRT, Buschke Selective Reminding Test; BVMT-R, Brief Visuospatial Memory Test–Revised; CANTAB PRM, Cambridge Neuropsychological Test Automated Battery Pattern Recognition Memory; CVLT, California Verbal Learning Test; HVLT-R, Hopkins Verbal Learning Test–Revised; RBANS, Repeatable Battery for the Assessment of Neuropsychological Status; RCFT, Rey-Osterrieth Complex Figure Test; SMD, standardized mean difference; SVLT, Serial Verbal Learning Task; TMT-A, Trail Making Test Part A; WAIS, Wechsler Adult Intelligence Scale; WMS, Wechsler Memory Scale.

**Figure 3.**
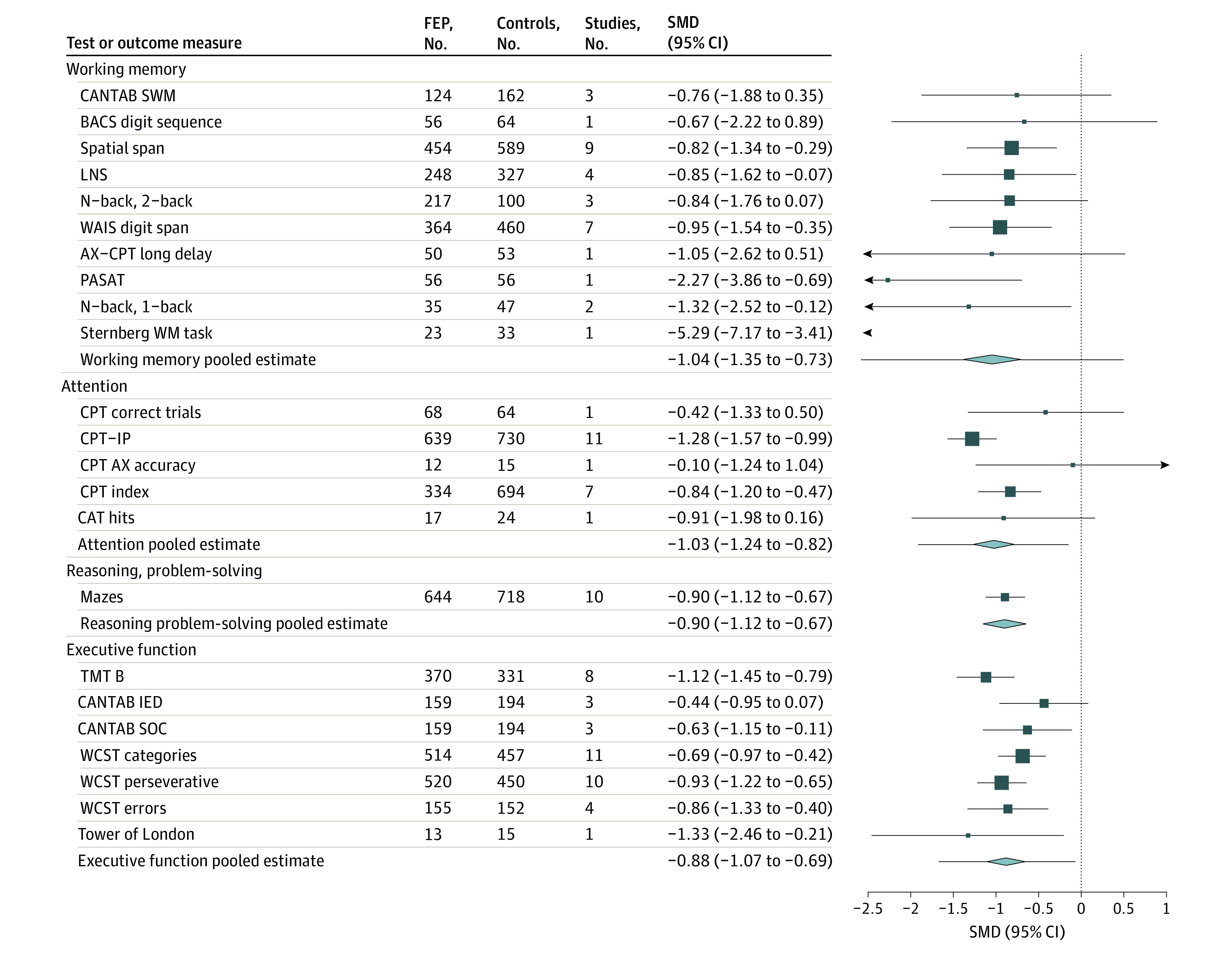
Combined Forest Plot of Mean Differences in Cognitive Ability for Working Memory, Attention, Reasoning/Problem Solving, and Executive Function Domains in Drug-Naive Patients With First-Episode Psychosis (FEP) Compared With Controls The lines extending from the pooled estimates indicate prediction interval. BACS indicates Brief Assessment of Cognition in Schizophrenia; CANTAB IED, Cambridge Neuropsychological Test Automated Battery intradimensional/extradimensional set shifting; CANTAB SOC, CANTAB Stockings of Cambridge; CANTAB SWM, CANTAB Spatial Working Memory; CPT, Continuous Performance Test; CPT-IP, Continuous Performance Test–Identical Pairs; LNS, Letter Number Sequencing; PASAT, Paced Auditory Serial Addition Test; SMD, standardized mean difference; TMT-B, Trail Making Test Part B; WAIS, Wechsler Adult Intelligence Scale; WCST, Wisconsin Card Sorting Test.

Individuals with FEP performed significantly worse than control individuals across all cognitive domains, with large effect sizes ranging from −0.88 to −1.16 (Figure 2 and Figure 3). In order of magnitude, the effect was largest for speed of processing (Hedges *g* = −1.16; 95% CI, −1.35 to −0.98), followed by verbal learning (Hedges *g* = −1.08; 95% CI, −1.28 to −0.88), visual learning (Hedges *g* = −1.05; 95% CI, −1.27 to −0.82), working memory (Hedges *g* = −1.04; 95% CI, −1.35 to −0.73), attention (Hedges *g* = −1.03; 95% CI, −1.24 to −0.82), reasoning/problem solving (Hedges *g* = −0.90; 95% CI, −1.12 to −0.68), and executive function (Hedges *g* = −0.88; 95% CI, −1.07 to −0.69). Study-level forest plots are available in eFigures 1 through 7 in Supplement 1.

### Heterogeneity, Study Quality, Publication Bias, and Meta-Regression

There was substantial heterogeneity in effect sizes. All cognitive domains displayed *I*^2^ estimates above 70%, suggesting that the variance in observed effects was mostly due to differences in true effects and not sampling error.^31^ Prediction intervals were large, indicating that the mean group difference could vary substantially depending on sample (eTable 12 in Supplement 1). Quality rating of the studies ranged from 3 to 8 (mean = 6.1, median = 6). Publication biases are reported in eTable 12 and eFigures 15 to 21 in Supplement 1. Meta-regressions revealed no strong effects of the potential moderators (age, sex, education, publication year, and study quality) that were assessed (eTable 15 in Supplement 1).

### Variability of Cognitive Functioning in Antipsychotic Drug–Naive Patients With FEP Compared With Controls

**Figure 4.**
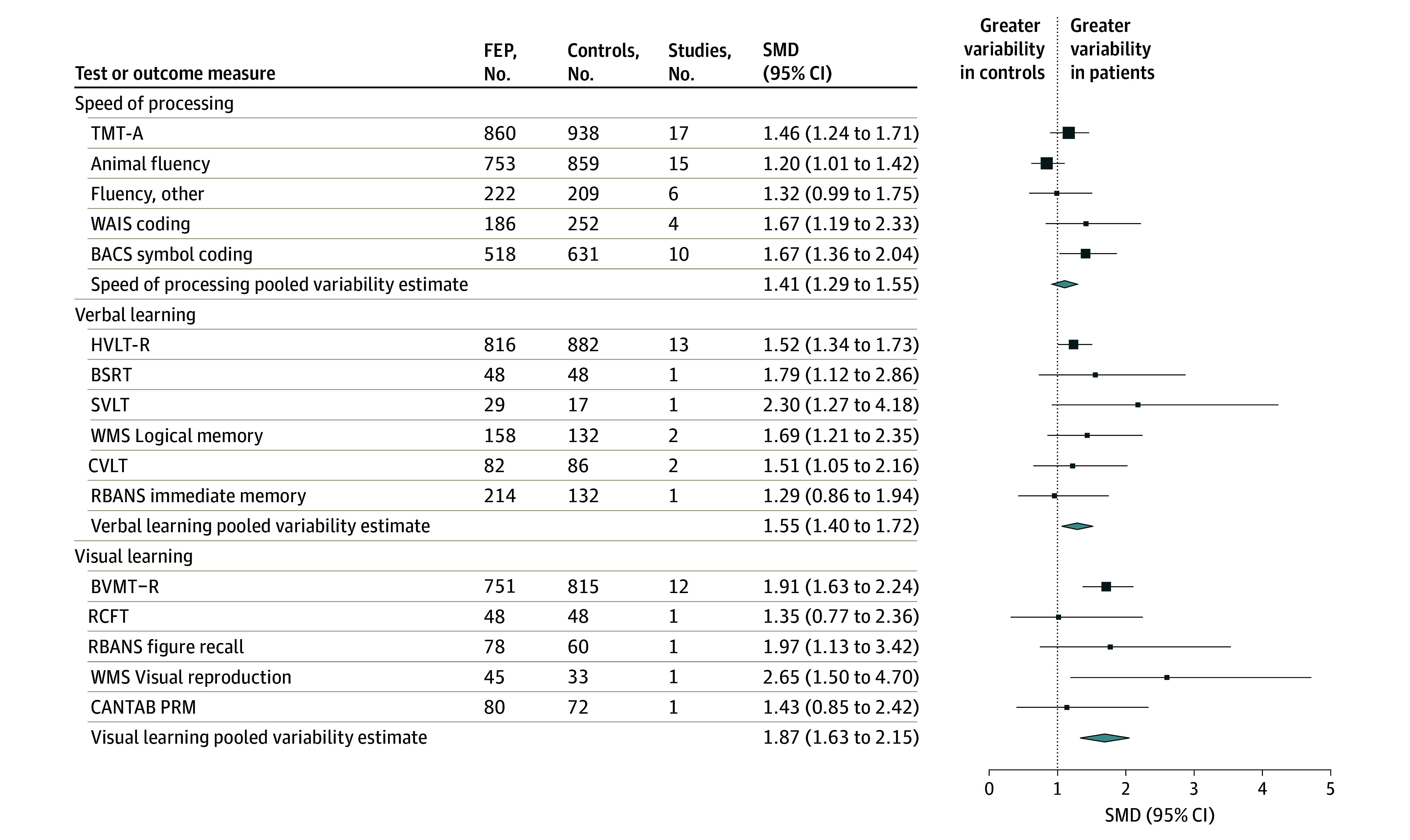
Combined Forest Plot of Within-Group Variability in Cognitive Ability for Speed of Processing, Verbal Learning, and Visual Learning Domains in Drug-Naive Patients With First-Episode Psychosis (FEP) Compared With Controls BACS indicates Brief Assessment of Cognition in Schizophrenia; BSRT, Buschke Selective Reminding Test; BVMT-R, Brief Visuospatial Memory Test-Revised; CANTAB PRM, Cambridge Neuropsychological Test Automated Battery Pattern Recognition Memory; CVLT, California Verbal Learning Test; HVLT-R, Hopkins Verbal Learning Test–Revised; RBANS, Repeatable Battery for the Assessment of Neuropsychological Status; RCFT, Rey-Osterrieth Complex Figure Test; SMD, standardized mean difference; SVLT, Serial Verbal Learning Task; TMT-A, Trail Making Test Part A; WAIS, Wechsler Adult Intelligence Scale; WMS, Wechsler Memory Scale.

**Figure 5.**
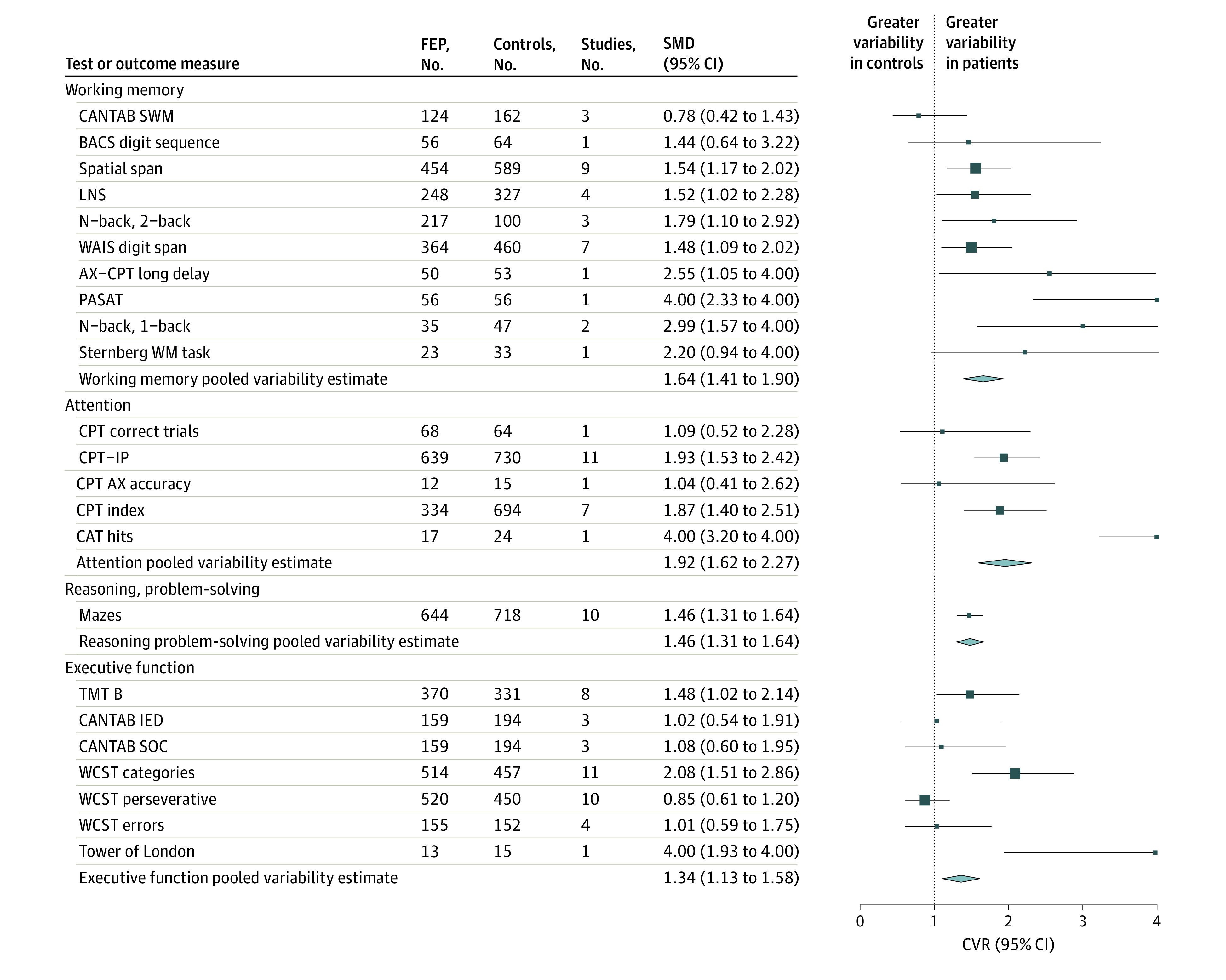
Combined Forest Plot of Within-Group Variability in Cognitive Ability for Working Memory, Attention, Reasoning/Problem Solving, and Executive Function Domains in Drug-Naive Patients With First-Episode Psychosis (FEP) Compared With Controls BACS indicates Brief Assessment of Cognition in Schizophrenia; CANTAB IED, Cambridge Neuropsychological Test Automated Battery intradimensional/extradimensional set shifting; CANTAB SOC, CANTAB Stockings of Cambridge; CANTAB SWM, CANTAB Spatial Working Memory; CPT, Continuous Performance Test; CPT-IP, Continuous Performance Test–Identical Pairs; CVR, coefficient of variation ratio; LNS, Letter Number Sequencing; PASAT, Paced Auditory Serial Addition Test; TMT-B, Trail Making Test Part B; WAIS, Wechsler Adult Intelligence Scale; WCST, Wisconsin Card Sorting Test.

The patients showed significantly greater within-group variability in cognitive performance compared with the controls. CVR values ranged between 1.34 to 1.92 (Figure 4 and Figure 5), meaning that variability in the patient group was about 30% as large to almost twice as large as that of the controls. In order of magnitude, the largest CVR was obtained for the attention domain (CVR = 1.92; 95% CI, 1.62-2.27), followed by visual learning (CVR = 1.87; 95% CI, 1.63-2.15), working memory (CVR = 1.61; 95% CI, 1.37-1.90), verbal learning (CVR = 1.55; 95% CI, 1.40-1.72), reasoning/problem solving (CVR = 1.46; 95% CI, 1.31-1.64), speed of processing (CVR = 1.43; 95% CI, 1.27-1.61), and executive function (CVR = 1.34; 95% CI, 1.13-1.58). Sensitivity analyses of within-group variability are available in eTable 14 in Supplement 1 as well as additional study-level forest plots of CVR in eFigures 8 through 14 in Supplement 1.

## Discussion

Results of this updated systematic review and meta-analysis supported the presence of substantial cognitive impairment in patients experiencing their FEP, before antipsychotic treatment has been introduced. As a group, patients underperformed compared with controls, with similar effect sizes, across all cognitive domains. Furthermore, we, for the first time (to our knowledge), performed a comprehensive meta-analytic comparison of within-group variability, demonstrating a greater variability in cognitive performance within the patient group compared with the healthy controls across all domains.

The present synthesis incorporated more than twice as many studies and participants as the previous meta-analysis on antipsychotic drug–naive individuals with FEP^11^ and was conducted using updated reporting standards and meta-analytic methods. Inclusion criteria followed the same principles, apart from a stricter selection in regard to DUP as we wanted to characterize the onset of the disorder and not the chronic effects of unmedicated psychosis. The updated literature search yielded some additional differences in study characteristics where a majority of studies (eTable 11 in Supplement 1) used the MCCB, and one-half of the new studies (eTable 11 in Supplement 1) included were conducted in 1 country (China) as compared with 25% of included studies in the previous meta-analysis.^11^ Effects detected in the current study were slightly larger (a difference of 0.08-0.33 in Cohen *d*). There are several possible explanations, including increased harmonization across studies and more reliable psychometrics, although meta-regression of publication year failed to show significance. The results are similar to those reported in chronic schizophrenia samples^1,2,3^ and are slightly larger compared with those observed in medicated FEP.^12,13^ Hence, our findings corroborate the notion of cognitive impairment as a central feature already present in the early stages of psychotic disorders,^32,33^ before the introduction of antipsychotic medication.

Similar to the previous meta-analysis, we observed a large proportion of variability between studies, where *I*^2^ values and prediction intervals show that the mean effect size can vary considerably depending on the sample. Meta-regressions did not reveal any strong effects of our preregistered potential moderators. However, recruiting antipsychotic drug–naive individuals with FEP for research is notoriously complicated, potentially resulting in different screening and recruitment procedures across studies, which could increase variability between studies.

Using CVR as an outcome, we found greater within-group variability in the patient group compared with the control group, across all cognitive domains (eDiscussion in Supplement 1). This indicates a wider distribution of cognitive ability in those with FEP compared with the normal population, meaning that some may experience severe cognitive problems and some may be relatively spared or may even be performing above average. The results can be viewed as support for the suggestion that patients with psychotic disorders can be divided into different cognitive subgroups,^16,17^ which may, in turn, share underlying biological disease mechanisms.^34^

Several lines of data indicate that cognitive impairment in psychotic disorders typically starts years before psychosis onset^8,9,10^; however, it is not clear whether the transition from the CHR-P or prodromal stage to a psychotic syndrome is associated with further cognitive deterioration.^35^ In a meta-analysis by Catalan and colleagues^36^ of cognitive function in those at CHR-P compared with healthy controls, the effect sizes obtained were in the medium range (−0.39 to −0.51), ie, considerably smaller than our results in patients with FEP. At the same time, the variability estimates^19^ were consistently lower (CVR range, 1.15-1.45 vs 1.34-1.94). Given that individuals at CHR-P represent a heterogeneous group where only a minority of individuals will go on to develop psychosis, less severe deficits on a group level are to be expected, whereas it may be seen as more surprising that cognitive variability is lower than in FEP. In addition to the individuals at CHR-P who transition to psychosis, the FEP group is made up of the estimated 10% who do not experience a prodrome^37,38^ (possibly adding more preserved cognitive ability) as well as those individuals who refuse to seek help before emergency services are needed^39,40^ (possibly adding more impaired individuals). Although interpreting cross-sectional data in a longitudinal context should be done with caution, it could be speculated that the increased variability may be a consequence of this addition of more spared, and more impaired, groups of patients. A further explanation could be that within the FEP group, there are individuals who experience considerably more cognitive decline than others during the transition to psychosis, resulting in this even wider distribution of cognitive performance (as compared with controls). This lends indirect support to the idea of different cognitive trajectories leading up to and during the transition to psychosis; however, longitudinal studies are needed to address these questions.

### Limitations

There are several limitations to address. A general concern is cognitive testing of unmedicated individuals with psychosis. This procedure provides data free from possible confounding effects of medication but may be associated with other factors affecting the test results, such as sleep deprivation, lack of motivation, and disruptive psychotic symptoms (although most studies fail to find a strong association between cognition and psychotic symptoms^35^). Regarding the CVR analysis, this measure depends not only on the performance and variance of the patient group but also on the characteristics of the control group. Controls who volunteer for research could theoretically be a selected group with less variability, meaning that their similarity would inflate the variability of the patient group. In the quality assessment of studies, the recruitment procedure for controls was scrutinized, and the majority of studies were reported using community controls. This would indicate that most researchers have attempted to address this issue. Furthermore, CVR values may have been influenced by the factors listed previously regarding the testing of individuals not taking medication, potentially adding to the degree of variability. Psychometric features of specific tasks used may also have contributed to our results, and tasks with lower reliability would likely result in more variability. Finally, we aimed to study cognitive function early in the psychotic illness process and, therefore, limited inclusion to studies with a mean sample DUP of 5 years or less (thereby excluding, eg, chronically unmedicated samples). However, a subgroup of the studies included did not report DUP. This is a possible limitation, although all studies included defined their sample as having FEP, and the mean age of nearly all samples would indicate a DUP of less than 5 years.

## Conclusions

Results of this systematic review and meta-analysis identified cognitive dysfunction already present at the onset of psychotic illness and before initiation of antipsychotic treatment and, for the first time (to our knowledge), revealed higher variability between individuals with FEP compared with controls. The results highlight the importance of cognitive assessments in clinical practice, in order to identify individuals at risk for poor functional outcome, and to select individuals for cognitive remediation,^41,42^ as part of a precision medicine approach.

## Notes

Supplement 1. **eTable 1.** PRISMA Statement and Checklist **eTable 2.** MOOSE Checklist **eMethods 1.** Search Terms Used in Literature Search **eMethods 2.** Discrepancies Between Cognitive Domains and Outcome Measures in the Present Analysis Compared With 2014 Meta-Analysis **eMethods 3.** KaSP Cohort—Methods, Analysis, and Results **eTable 3.** Demographic Data on Drug-Naive FEP and Controls Included in the Meta-Analysis **eTable 4.** Cognitive Data on Drug-Naive FEP and Controls Included in the Meta-Analysis **eTable 5.** Demographic Data on Full KaSP Sample and Controls **eTable 6.** Cognitive Data on Full KaSP Sample and Controls **eTable 7.** Correlations Between Cognitive Scores and Clinical Assessments (Full KaSP Sample) **eTable 8.** Exploratory Correlations Between Cognitive Subtests and GAF (Full KaSP Sample) **eMethods 4.** Neurocognitive Tests and Outcome Measures per Domain **eTable 9.** List of All Tests and Outcome Measures Used **eTable 10.** Studies That Were Assumed to Overlap **eMethods 5.** Methods for Pooling Nonindependent Cognitive Tasks **eMethods 6.** Modified Newcastle Ottawa Scale **eMethods 7.** Regarding the CVR Measure **eMethods 8.** Regarding Meta-Regressions
eDiscussion
**eTable 11.** Characteristics of Included Studies **eTable 12.** Meta-Analysis Results, Mean Differences **eTable 13.** Meta-Analysis Results, Within-Group Variability **eTable 14.** Meta-Analysis Results, Within-Group Variability (Sensitivity Analysis With Separate Analysis for Negative Outcome Measures) **eTable 15.** Meta-Regressions **eFigure 1.** Forest Plot for Processing Speed, Mean Differences **eFigure 2.** Forest Plot for Verbal Learning, Mean Differences **eFigure 3.** Forest Plot for Visual Learning, Mean Differences **eFigure 4.** Forest Plot for Working Memory, Mean Differences **eFigure 5.** Forest Plot for Attention, Mean Differences **eFigure 6.** Forest Plot for Reasoning/Problem-Solving, Mean Differences **eFigure 7.** Forest Plot for Executive Function, Mean Differences **eFigure 8.** Forest Plot for Processing Speed, CVR Variability **eFigure 9.** Forest Plot for Verbal Learning, CVR Variability **eFigure 10.** Forest Plot for Visual Learning, CVR Variability **eFigure 11.** Forest Plot for Working Memory, CVR Variability **eFigure 12.** Forest Plot for Attention, CVR Variability **eFigure 13.** Forest Plot for Reasoning/Problem-Solving, CVR Variability **eFigure 14.** Forest Plot for Executive Function, CVR Variability **eFigure 15.** Funnel Plot for Processing Speed **eFigure 16.** Funnel Plot for Verbal Learning **eFigure 17.** Funnel Plot for Visual Learning **eFigure 18.** Funnel Plot for Working Memory **eFigure 19.** Funnel Plot for Attention **eFigure 20.** Funnel Plot for Reasoning/Problem-Solving **eFigure 21.** Funnel Plot for Executive Function
eReferences

Supplement 2.
Data Sharing Statement.
