# Supplementary material for: Cognitive Function and Variability in Antipsychotic Drug–Naive Patients With First-Episode Psychosis: A Systematic Review and Meta-Analysis
Source: JAMA Psychiatry. 2024 Feb 28;81(5):468–76. doi: 10.1001/jamapsychiatry.2024.0016 (PMC10902783; doi:10.1001/jamapsychiatry.2024.0016)
Supplement: Supplement 1. — eTable 1. PRISMA Statement and Checklist eTable 2. MOOSE Checklist eMethods 1. Search Terms Used in Literature Search eMethods 2. Discrepancies Between Cognitive Domains and Outcome Measures in the Present Analysis Compared With 2014 Meta-Analysis eMethods 3. KaSP Cohort—Methods, Analysis, and Results eTable 3. Demographic Data on Drug-Naive FEP and Controls Included in the Meta-Analysis eTable 4. Cognitive Data on Drug-Naive FEP and Controls Included in the Meta-Analysis eTable 5. Demographic Data on Full KaSP Sample and Controls eTable 6. Cognitive Data on Full KaSP Sample and Controls eTable 7. Correlations Between Cognitive Scores and Clinical Assessments (Full KaSP Sample) eTable 8. Exploratory Correlations Between Cognitive Subtests and GAF (Full KaSP Sample) eMethods 4. Neurocognitive Tests and Outcome Measures per Domain eTable 9. List of All Tests and Outcome Measures Used eTable 10. Studies That Were Assumed to Overlap eMethods 5. Methods for Pooling Nonindependent Cognitive Tasks eMethods 6. Modified Newcastle Ottawa Scale eMethods 7. Regarding the CVR Measure eMethods 8. Regarding Meta-Regressions eDiscussion eTable 11. Characteristics of Included Studies eTable 12. Meta-Analysis Results, Mean Differences eTable 13. Meta-Analysis Results, Within-Group Variability eTable 14. Meta-Analysis Results, Within-Group Variability (Sensitivity Analysis With Separate Analysis for Negative Outcome Measures) eTable 15. Meta-Regressions eFigure 1. Forest Plot for Processing Speed, Mean Differences eFigure 2. Forest Plot for Verbal Learning, Mean Differences eFigure 3. Forest Plot for Visual Learning, Mean Differences eFigure 4. Forest Plot for Working Memory, Mean Differences eFigure 5. Forest Plot for Attention, Mean Differences eFigure 6. Forest Plot for Reasoning/Problem-Solving, Mean Differences eFigure 7. Forest Plot for Executive Function, Mean Differences eFigure 8. Forest Plot for Processing Speed, CVR Variability eFigure 9. Forest Plot for Verbal Learning, CV [file jamapsychiatry-e240016-s001.pdf]

Supplementary Online Content

Lee M, Cernvall M, Borg J, et al. Cognitive function and variability in antipsychotic drug–naive patients with first-episode psychosis: a systematic review and meta-analysis. *JAMA Psychiatry*. Published online February 28, 2024. doi:10.1001/jamapsychiatry.2024.0016

eTable 1. PRISMA Statement and Checklist

eTable 2. MOOSE Checklist

eMethods 1. Search Terms Used in Literature Search

eMethods 2. Discrepancies Between Cognitive Domains and Outcome Measures in the Present Analysis Compared With 2014 Meta-Analysis

eMethods 3. KaSP Cohort—Methods, Analysis, and Results

eTable 3. Demographic Data on Drug-Naive FEP and Controls Included in the Meta-Analysis

eTable 4. Cognitive Data on Drug-Naive FEP and Controls Included in the Meta-Analysis

eTable 5. Demographic Data on Full KaSP Sample and Controls

eTable 6. Cognitive Data on Full KaSP Sample and Controls

eTable 7. Correlations Between Cognitive Scores and Clinical Assessments (Full KaSP Sample)

eTable 8. Exploratory Correlations Between Cognitive Subtests and GAF (Full KaSP Sample)

eMethods 4. Neurocognitive Tests and Outcome Measures per Domain

eTable 9. List of All Tests and Outcome Measures Used

eTable 10. Studies That Were Assumed to Overlap

eMethods 5. Methods for Pooling Nonindependent Cognitive Tasks

© 2024 Lee M et al. *JAMA Psychiatry.*

eMethods 6. Modified Newcastle Ottawa Scale

eMethods 7. Regarding the CVR Measure

eMethods 8. Regarding Meta-Regressions

eDiscussion.

eTable 11. Characteristics of Included Studies

eTable 12. Meta-Analysis Results, Mean Differences

eTable 13. Meta-Analysis Results, Within-Group Variability

eTable 14. Meta-Analysis Results, Within-Group Variability (Sensitivity Analysis With Separate Analysis for Negative Outcome Measures)

eTable 15. Meta-Regressions

eFigure 1. Forest Plot for Processing Speed, Mean Differences

eFigure 2. Forest Plot for Verbal Learning, Mean Differences

eFigure 3. Forest Plot for Visual Learning, Mean Differences

eFigure 4. Forest Plot for Working Memory, Mean Differences

eFigure 5. Forest Plot for Attention, Mean Differences

eFigure 6. Forest Plot for Reasoning/Problem-Solving, Mean Differences

eFigure 7. Forest Plot for Executive Function, Mean Differences

eFigure 8. Forest Plot for Processing Speed, CVR Variability

eFigure 9. Forest Plot for Verbal Learning, CVR Variability

eFigure 10. Forest Plot for Visual Learning, CVR Variability

eFigure 11. Forest Plot for Working Memory, CVR Variability
© 2024 Lee M et al. *JAMA Psychiatry.*

eFigure 12. Forest Plot for Attention, CVR Variability

eFigure 13. Forest Plot for Reasoning/Problem-Solving, CVR Variability

eFigure 14. Forest Plot for Executive Function, CVR Variability

eFigure 15. Funnel Plot for Processing Speed

eFigure 16. Funnel Plot for Verbal Learning

eFigure 17. Funnel Plot for Visual Learning

eFigure 18. Funnel Plot for Working Memory

eFigure 19. Funnel Plot for Attention

eFigure 20. Funnel Plot for Reasoning/Problem-Solving

eFigure 21. Funnel Plot for Executive Function

eReferences

© 2024 Lee M et al. *JAMA Psychiatry.*

eTable 1. PRISMA statement and checklist

Section and Topic	Item #	Checklist item	Location where item is reported
**TITLE**			
Title	1	Identify the report as a systematic review.	Cover page
**ABSTRACT**			
Abstract	2	See the PRISMA 2020 for Abstracts checklist.	Abstract
**INTRODUCTION**			
Rationale	3	Describe the rationale for the review in the context of existing knowledge.	Introduction
Objectives	4	Provide an explicit statement of the objective(s) or question(s) the review addresses.	Introduction
**METHODS**			
Eligibility criteria	5	Specify the inclusion and exclusion criteria for the review and how studies were grouped for the syntheses.	Method
Information sources	6	Specify all databases, registers, websites, organisations, reference lists and other sources searched or consulted to identify studies. Specify the date when each source was last searched or consulted.	Method, eMethods 1
Search strategy	7	Present the full search strategies for all databases, registers and websites, including any filters and limits used.	eMethods 1
Selection process	8	Specify the methods used to decide whether a study met the inclusion criteria of the review, including how many reviewers screened each record and each report retrieved, whether they worked independently, and if applicable, details of automation tools used in the process.	Method
Data collection process	9	Specify the methods used to collect data from reports, including how many reviewers collected data from each report, whether they worked independently, any processes for obtaining or confirming data from study investigators, and if applicable, details of automation tools used in the process.	Method
Data items	10a	List and define all outcomes for which data were sought. Specify whether all results that were compatible with each outcome domain in each study were sought (e.g. for all measures, time points, analyses), and if not, the methods used to decide which results to collect.	Method, eMethods 4, eTable 9
	10b	List and define all other variables for which data were sought (e.g. participant and intervention characteristics, funding sources). Describe any assumptions made about any missing or unclear information.	eTable 11
Study risk of bias assessment	11	Specify the methods used to assess risk of bias in the included studies, including details of the tool(s) used, how many reviewers assessed each study and whether they worked independently, and if applicable, details of automation tools used in the process.	Method
Effect measures	12	Specify for each outcome the effect measure(s) (e.g. risk ratio, mean difference) used in the synthesis or presentation of results.	Method
Synthesis methods	13a	Describe the processes used to decide which studies were eligible for each synthesis (e.g. tabulating the study intervention characteristics and comparing against the planned groups for each synthesis (item #5)).	Method
	13b	Describe any methods required to prepare the data for presentation or synthesis, such as handling of missing summary statistics, or data conversions.	Method, eMethods 5-6

© 2024 Lee M et al. *JAMA Psychiatry.*

Section and Topic	Item #	Checklist item	Location where item is reported
	13c	Describe any methods used to tabulate or visually display results of individual studies and syntheses.	-
	13d	Describe any methods used to synthesize results and provide a rationale for the choice(s). If meta-analysis was performed, describe the model(s), method(s) to identify the presence and extent of statistical heterogeneity, and software package(s) used.	Method
	13e	Describe any methods used to explore possible causes of heterogeneity among study results (e.g. subgroup analysis, meta-regression).	Method, eTable 15
	13f	Describe any sensitivity analyses conducted to assess robustness of the synthesized results.	Method, eMethods 6, eTable14
Reporting bias assessment	14	Describe any methods used to assess risk of bias due to missing results in a synthesis (arising from reporting biases).	Method
Certainty assessment	15	Describe any methods used to assess certainty (or confidence) in the body of evidence for an outcome.	Method, eMethods 6
**RESULTS**			
Study selection	16a	Describe the results of the search and selection process, from the number of records identified in the search to the number of studies included in the review, ideally using a flow diagram.	Figure 1
	16b	Cite studies that might appear to meet the inclusion criteria, but which were excluded, and explain why they were excluded.	-
Study characteristics	17	Cite each included study and present its characteristics.	eTable 11
Risk of bias in studies	18	Present assessments of risk of bias for each included study.	eTable 11
Results of individual studies	19	For all outcomes, present, for each study: (a) summary statistics for each group (where appropriate) and (b) an effect estimate and its precision (e.g. confidence/credible interval), ideally using structured tables or plots.	eFigures 1-14
Results of syntheses	20a	For each synthesis, briefly summarise the characteristics and risk of bias among contributing studies.	Results
	20b	Present results of all statistical syntheses conducted. If meta-analysis was done, present for each the summary estimate and its precision (e.g. confidence/credible interval) and measures of statistical heterogeneity. If comparing groups, describe the direction of the effect.	Results
	20c	Present results of all investigations of possible causes of heterogeneity among study results.	Results
	20d	Present results of all sensitivity analyses conducted to assess the robustness of the synthesized results.	eTable14
Reporting biases	21	Present assessments of risk of bias due to missing results (arising from reporting biases) for each synthesis assessed.	eTable 12, eFigures 15-21

© 2024 Lee M et al. *JAMA Psychiatry.*

Section and Topic	Item #	Checklist item	Location where item is reported
Certainty of evidence	22	Present assessments of certainty (or confidence) in the body of evidence for each outcome assessed.	Results
**DISCUSSION**			
Discussion	23a	Provide a general interpretation of the results in the context of other evidence.	Discussion
	23b	Discuss any limitations of the evidence included in the review.	Discussion
	23c	Discuss any limitations of the review processes used.	Discussion
	23d	Discuss implications of the results for practice, policy, and future research.	Discussion
**OTHER INFORMATION**			
Registration and protocol	24a	Provide registration information for the review, including register name and registration number, or state that the review was not registered.	Method
	24b	Indicate where the review protocol can be accessed, or state that a protocol was not prepared.	Method
	24c	Describe and explain any amendments to information provided at registration or in the protocol.	-
Support	25	Describe sources of financial or non-financial support for the review, and the role of the funders or sponsors in the review.	
Competing interests	26	Declare any competing interests of review authors.	
Availability of data, code and other materials	27	Report which of the following are publicly available and where they can be found: template data collection forms; data extracted from included studies; data used for all analyses; analytic code; any other materials used in the review.	Method

© 2024 Lee M et al. *JAMA Psychiatry.*

eTable 2. MOOSE checklist

Item No	Recommendation	Reported on Page No
Reporting of background should include		
1	Problem definition	6-8
2	Hypothesis statement	-
3	Description of study outcome(s)	9, Suppl 8, 13-14
4	Type of exposure or intervention used	8-9
5	Type of study designs used	PROSPERO
6	Study population	8
Reporting of search strategy should include		
7	Qualifications of searchers (eg, librarians and investigators)	Title page
8	Search strategy, including time period included in the synthesis and key words	8, Suppl 8
9	Effort to include all available studies, including contact with authors	8-9
10	Databases and registries searched	8
11	Search software used, name and version, including special features used (eg, explosion)	8
12	Use of hand searching (eg, reference lists of obtained articles)	8-9
13	List of citations located and those excluded, including justification	8, Fig 1
14	Method of addressing articles published in languages other than English	8
15	Method of handling abstracts and unpublished studies	8
16	Description of any contact with authors	8-9
Reporting of methods should include		
17	Description of relevance or appropriateness of studies assembled for assessing the hypothesis to be tested	8
18	Rationale for the selection and coding of data (eg, sound clinical principles or convenience)	9
19	Documentation of how data were classified and coded (eg, multiple raters, blinding and interrater reliability)	9
20	Assessment of confounding (eg, comparability of cases and controls in studies where appropriate)	11
21	Assessment of study quality, including blinding of quality assessors, stratification or regression on possible predictors of study results	10
22	Assessment of heterogeneity	10
23	Description of statistical methods (eg, complete description of fixed or random effects models, justification of whether the chosen models account for predictors of study results, dose-response models, or cumulative meta-analysis) in sufficient detail to be replicated	10-11 (incl Github ref), Suppl 19, 22
24	Provision of appropriate tables and graphics	Fig 2-3, Suppl

© 2024 Lee M et al. *JAMA Psychiatry.*

		Tables 12-13 & 15 Suppl Figs 1-14
Reporting of results should include		
25	Graphic summarizing individual study estimates and overall estimate	Figs 2-3
26	Table giving descriptive information for each study included	Suppl eTable 11
27	Results of sensitivity testing (eg, subgroup analysis)	Suppl eTable 14
28	Indication of statistical uncertainty of findings	12-13
Reporting of discussion should include		
29	Quantitative assessment bias	10, Suppl eTable 12, Suppl Figs 15-21
30	Justification for exclusion	8
31	Assessment of quality of included studies	10, Suppl eMethods 6, eTable 11
Reporting of conclusions should include		
32	Consideration of alternative explanations for observed results	13-16
33	Generalization of the conclusions	14-16
34	Guidelines for future research	16
35	Disclosure of funding source	17

© 2024 Lee M et al. *JAMA Psychiatry.*

eMethods 1. Search terms used in literature search

Full search term used:

((((cognition OR cognitive OR neurocognitive OR neuropsychological OR neuropsychologic OR neurocognition)) AND ((psychosis OR psychotic OR schizophrenia))) AND ((drug naïve OR drug-naïve OR never treated OR never-treated OR neuroleptic naïve OR neuroleptic-naïve OR anti- psychotic naïve OR antipsychotic-naïve OR never medicated OR never- medicated OR treatment naïve OR treatment-naïve))) AND (("2012"[Date - Publication] : "3000"[Date - Publication]))

The last search was performed on September 15th 2022.

eMethods 2. Discrepancies between cognitive domains and outcome measures in the present analysis compared to 2014 meta-analysis

Cognitive tests were grouped in a similar way as in the meta-analysis from 2014[1] following the domains of the MCCB except for social cognition (considered to be beyond the scope of this review). In our previous meta-analysis, the domain of executive function was added to replace the MATRICS domain reasoning and problem solving, as no studies reported on this. In the current analysis, the domain of Executive Function was retained, and the domain Reasoning and Problem Solving, as assessed by the Mazes test of the MCCB, was added.

The tests and outcome measures used and reported in the meta-analysis from 2014[1] were also included in the current synthesis, except for when the studies did not meet inclusion criteria (1 study where DUP was too long [study McCreadie et al., 1997[2], mean DUP = 15 years], 1 study with overlapping sample [study Barch et al., 2003[3], overlapping with Richard et al., 2013[4]]). Furthermore, in one case we did not include all of the outcome measures reported, as some of them were included in larger samples in the updated search (Andersen et al., 2013[5], all CANTAB tests were included in Jessen et al., 2019[6])

In some cases we chose to exclude rare and overlapping outcome measures, when several other outcome measures were reported from the same study, in order to increase harmonization. This involved tests of attention, where our intention was to focus on overall measures of CPT-performance (such as d' or A'), and other outcome measures were omitted (Finkelstein et al., 1997: the outcome measures omission errors and commission errors dropped, He et al., 2013: the outcome measure mean latency from the test CANTAB RVP dropped, Salgado-Pineda et al., 2003: the outcome measures omission errors and commission errors from the CPT-IP test dropped, Wang et al., 2007: the outcome measure hit rate from the test CPT-37 version dropped). Furthermore, in Hong et al., 2002 the CPT-like test Vigilance (outcome measure: hits) was excluded as the study reported a very similar outcome measure (Continuous Attention Test; CAT)

Finally we also added some outcome measures that were not in the meta-analysis from 2014, as they mapped on to other outcome measures in abundance from the new literature search: Hill, Schuepbach et al., 2004: Fluency (COWAT), WCST percentage perseverative errors

© 2024 Lee M et al. *JAMA Psychiatry.*

eMethods 3. KaSP cohort – methods, analysis and results

Methods

Project

The Karolinska Schizophrenia Project (KaSP) is an ongoing research project recruiting first-episode psychosis patients (FEP) in the greater Stockholm area since 2011. It was approved by the Regional Ethics Committee in Stockholm (diary number: 2010/879-31-1). Individuals with FEP are recruited from health care settings (in-patient psychiatric wards, out-patient clinics) and control participants are recruited via advertisement, through an online portal listing available research projects. After receiving a full description of the study, participants provide written informed consent, in accordance with the Declaration of Helsinki.

Participants

FEP were included if they met the criteria for any disorder in the DSM-IV chapter on Psychotic Disorders, assessed by an MD or clinical psychologist using the Structured Clinical Interview for DSM-IV (SCID-I) and had less than four weeks of exposure to antipsychotic medication. Control participants (n = 64) are matched on age and gender and recruited through advertisement. Exclusion criteria for FEP and controls were current or history of abuse of alcohol or illegal drugs, severe somatic illness or neurological disorder (ruled out through medical history, clinical examination, laboratory tests, and brain magnetic resonance imaging). Further exclusion criteria for control participants were previous or current psychiatric illness assessed by Mini International Neuropsychiatric Interview, lifetime use of anti-psychotics, or first-degree relatives with psychotic illness. For the current analysis of cognitive function individuals above the age of 45 were excluded (n = 2), making the final sample 64 controls and 86 FEP. Approximately half (n= 42) of the FEP were drug-naïve to anti-psychotic medication and are included in the meta-analysis along with the controls. Data on the full sample are also presented here.

Examinations and clinical assessments

In KaSP, FEP and controls undergo several physical examinations and clinical assessments within 1-2 weeks of enrollment, including: structural magnetic resonance imaging (MRI) and resting state functional MRI, lumbar puncture, blood sampling, skin-biopsy, pre-pulse inhibition (PPI), cognitive testing, as well as positron emission tomography (PET) for a subset of participants. The clinical assessment of FEP include the Global Assessment of Functioning (GAF) and Clinical Global Impression (CGI), as well as the Positive and Negative Syndrome Scale (PANSS) to assess positive, negative and general psychotic symptoms.

Cognitive testing

Cognitive assessment consisted of the MATRICS MCCB and the Wisconsin Card Sorting Test. Testing was conducted by a clinical psychologist or a trained research nurse under the supervision of a clinical psychologist.

Statistical analysis

An analysis plan was discussed and decided within the analysis group before looking at data. Our aim was to:

1) Compare the whole FEP group to controls, as well as to compare the drug-naïve subgroup to controls.
2) Explore correlations between cognitive performance in FEP and clinical variables.

These two research questions (group comparison and correlation) were considered as different families of statistical tests and hence correction for multiple comparisons was performed for the two analyses separately. A power analysis was performed using previously published data. Based on this, group comparisons were conducted for all subtests of the MCCB, MCCB

© 2024 Lee M et al. *JAMA Psychiatry.*

neurocognitive composite, WCST perseverative errors and WCST categories completed. Furthermore, correlations were explored between the neurocognitive composite and the GAF as well as PANSS Negative symptoms.

Exploratory analyses (not corrected for multiple comparisons) were conducted between all separate subtests and GAF.

Correction for multiple comparison was done using the meff function[7] for the group comparisons, as the cognitive test scores are all intercorrelated, resulting in the new p-value = 0.0067. The correlation analysis was corrected using Bonferroni correction, resulting in a new p-value = 0.025.

Raw scores were used in analyses to compare performance of FEP and controls on the different outcome measures. A neurocognitive composite was created by adding the raw scores (TMT A scores were flipped so that higher values meant better performance) of all neurocognitive MCCB tests (excluding the Social Cognition test MSCEIT) and dividing the sum by the amount of tests. This composite score was used in the primary correlational analyses.

Results

Demographic and cognitive data, drug-naïve FEP only.

eTable 3, demographic data on drug-naïve FEP and controls in KaSP included in the meta-analysis

	FEP N = 42	HC N = 64	p-value
**Age**	26.7 (6.45)	27.05 (5.62)	0.801
**Gender male/female**	26/16	31/33	0.174
**Education**	13.7 (3.2) N = 34	15.00 (2.3)	0.044
**PANSS Positive**	18.7 (5.3)		
**PANSS Negative**	15.6 (6.3)		
**PANSS General**	35.7 (10.9)		
**PANSS Total**	70.1 (19.2)		
**GAF**	45.2 (13.1)		
**DUP**	10.1 (14.9) N = 38		

© 2024 Lee M et al. *JAMA Psychiatry.*

eTable 4, cognitive data on drug-naïve FEP and controls in KaSP included in the meta-analysis

	Drug-naïve FEP N = 42	HC N = 64	p-value	Effect size (95 % CI)
**TMT**	30.38 (11.9)	22.89 (7.9)	0.0006441	0.77 (0.37 – 1.18)
**BACS SC**	49.43 (11.7)	62.16 (10.5)	0.000000191	1.16 (0.73, 1.57)
**Fluency**	20.98 (5.7)	28.36 (7.1)	0.00000005632	1.12 (0.70, 1.54)
**LNS**	13.81 (3.1)	15.52 (2.8)	0.005263	0.59 (0.19, 0.99)
**Spatial Span**	16.39 (3.3)	18.00 (2.7)	0.009962	0.55 (0.15, 0.95)
**Mazes**	20.68 (5.3)	23.00 (3.8)	0.01769	0.52 (0.12, 0.92)
**HVLT-R**	23.88 (5.4)	28.19 (3.6)	0.00002484	0.98 (0.56, 1.38)
**BVMTR-R**	23.66 (6.7)	29.66 (4.8)	0.00000537	1.07 (0.65, 1.48)
**CPT-IP**	2.26 (0.6)	2.89 (0.5)	0.0000007496	1.17 (0.74, 1.59)
**Neurocognitive composite**	299.50 (35.4)	341.31 (27.9)	0.00000002019	1.35 (0.91, 1.78)
**MSCEIT***	89.51 (11.8) N = 42	97.27 (7.0)	0.0004465	0.85 (0.44, 1.27)
**WCST categories**	5.16 (1.6) N = 37	5.81 (0.8) N = 63	0.02228	0.57 (0.16, 0.99)
**WCST pers errors**	16.87 (14.0) N = 37	8.14 (6.09) N = 63	0.0007921	-0.89 (-1.32, -0.47)

*MSCEIT was not included in the meta-analysis, as social cognition was not one of the domains analyzed.

Demographic and cognitive data, full KaSP sample

eTable 5, demographic data on full KaSP sample and controls.

	FEP N = 86	HC N = 64	p-value
**Age**	27.90 (7.0)	27.05 (5.6)	0.412
**Gender male/female**	53/33	31/33	0.108
**Education**	14.01 (3.1)	15.00 (2.3)	0.034
**PANSS Positive**	18.33 (5.8)		
**PANSS Negative**	16.6 (6.7)		
**PANSS General**	36.6 (10.5)		
**PANSS Total**	71.5 (19.2)		
**GAF**	42.53 (13.4) N = 85		
**DUP**	9.92 (13.6) N = 71		

© 2024 Lee M et al. *JAMA Psychiatry.*

eTable 6, cognitive data on full KaSP sample and controls

	FEP N = 86	HC N = 64	p-value	Effect size (95 % CI)
**TMT**	34.08 (18.4)	22.89 (7.9)	0.000001534	0.75 (0.42, 1.09)
**BACS SC**	46.85 (12.8)	62.16 (10.5)	0.0000000000002809	1.29 (0.93, 1.64)
**Fluency**	20.76 (5.8)	28.36 (7.1)	0.0000000002172	1.18 (0.83, 1.53)
**LNS**	12.80 (3.3)	15.52 (2.8)	0.0000002001	0.88 (0.54, 1.22)
**Spatial Span**	16.07 (2.9)	18.00 (2.7)	0.00004878	0.69 (0.35, 1.02)
**Mazes**	18.87 (6.5)	23.00 (3.8)	0.000003746	0.75 (0.41, 1.08)
**HVLT-R**	23.99 (5.27)	28.19 (3.6)	0.00000004539	0.90 (0.56, 1.24)
**BVMTR-R**	22.54 (7.1)	29.66 (4.8)	0.00000000001819	1.15 (0.80, 1.50)
**CPT-IP**	2.14 (0.7)	2.89 (0.5)	0.000000000001941	1.25 (0.89, 1.61)
**Neurocognitive**	291.24 (38.7)	341.31 (27.9)	0.000000000000001343	1.46 (1.09, 1.83)
**MSCEIT**	87.99 (11.1) N = 80	97.27 (7.0)	0.000000008914	0.98 (0.63, 1.33)
**WCST categories**	4.81 (1.87) N = 80	5.81 (0.8) N = 63	0.00003464	0.67 (0.33, 1.01)
**WCST pers errors**	16.41 (12.8) N = 80	8.14 (6.09) N = 63	0.000001263	-0.80 (-1.14, -0.45)

eTable 7, correlations between cognitive scores and clinical assessments (full KaSP sample)

	Clinical assessment	*r (95 % CI)*	p-value
**Neurocognitive composite**	GAF	.16 (-.06, .37)	0.168
**Neurocognitive composite**	PANSS Negative	-.27 (-.46, -.05)	0.016

eTable 8, exploratory correlations between cognitive subtests and GAF (full KaSP sample)

Cognitive test	Clinical assessment	*r (95 % CI)*	p-value
**TMT**	GAF	-.04 (-.25, .17)	0.696
**BACS SC**	GAF	.12 (-.10, .33)	0.273
**HVLT-R**	GAF	.05 (-.16, .26)	0.629
**Spatial Span**	GAF	.11 (-.10, .32)	0.306
**LNS**	GAF	.07 (-.15, .28)	0.547
**Mazes**	GAF	.18 (-.04, .38)	0.110
**BVMT-R**	GAF	.16 (-.06, .37)	0.143
**Fluency**	GAF	.04 (-.17, .26)	0.690
**CPT-IP**	GAF	-.02 (-.24, .21)	0.890

© 2024 Lee M et al. *JAMA Psychiatry.*

eMethods 4. Neurocognitive tests and outcome measures per domain

Certain neurocognitive outcome measures that are very similar and measure analogous cognitive abilities have been combined. In the table below (eTable 9) all tests and outcome measures used in the present analysis are listed (in the second column), and what they are referred to in figures and tables (in the third column).

eTable 9. List of all tests and outcome measures used

Neurocognitive domain	Test and outcome measure	Included in
**Processing speed**	▪ Trail Making Test - A<br>▪ Animal fluency<br>▪ FAS fluency<br>▪ Fluency actions<br>▪ Brief Assessment of Cognition in Schizophrenia Symbol - Coding<br>▪ Wechsler Adult Intelligence Scale - Coding	TMT A<br>Animal fluency<br>Fluency, other<br>Fluency, other<br>BACS SC<br>WAIS Coding
**Verbal learning**	▪ Hopkins Verbal Learning Test-Revised<br>▪ Buschke Selective Reminding Test<br>▪ Serial Verbal Learning Task<br>▪ California Verbal Learning Test<br>▪ Wechsler Memory Scale - Logical memory<br>▪ Repeatable Battery for the Assessment of Neuropsychological Status - Immediate memory	HVLT-R<br>BSRT<br>SVLT<br>CVLT<br>WMS Logical Memory<br>RBANS Immediate Memory
**Visual learning**	▪ Brief Visuospatial Memory Test-Revised<br>▪ Rey-Osterrieth Complex Figure Test<br>▪ Repeatable Battery for the Assessment of Neuropsychological Status - Figure Recall<br>▪ Wechsler Memory Scale - Visual reproduction<br>▪ Cambridge Neuropsychological Test Automated Battery - Pattern Recognition Memory test	BVMT-R<br>RCFT<br>RBANS Figure Recall<br>WMS Visual reproduction<br>CANTAB PRM
**Working memory**	▪ Cambridge Neuropsychological Test Automated Battery - Spatial Working Memory<br>▪ Brief Assessment of Cognition in Schizophrenia Symbol - Digit Sequence<br>▪ Wechsler Memory Scale - Spatial Span – 3rd ed.<br>▪ Letter Number Span<br>▪ N-back, 2-back<br>▪ Wechsler Adult Intelligence Scale - Digit Span<br>▪ AX-CPT long delay<br>▪ Paced Auditory Serial Addition Test<br>▪ N-back, 1-back<br>▪ Sternberg Working Memory task	CANTAB SWM<br>BACS digit sequence<br>Spatial Span<br>LNS<br>N-back, 2-back<br>WAIS digit span<br>AX-CPT long delay<br>PASAT<br>N-back, 1-back<br>Sternberg WM task
**Attention**	▪ Continuous Performance Test - Identical Pairs (CPT-IP)<br>▪ Continuous Performance Test – correct trials<br>▪ Continuous Performance Test – AX accuracy<br>▪ Cambridge Neuropsychological Test Automated Battery – Rapid Visual Information Processing: A<br>▪ Continuous Performance Test: d´<br>▪ Continuous Performance Test: A<br>▪ CAT hits	CPT-IP<br>CPT_correct trials<br>CPT_AX_accuracy<br>CPT_index<br>CPT_index<br>CPT_index<br>CAT_hits
**Reasoning/ problem solving**	▪ Mazes	Mazes

© 2024 Lee M et al. *JAMA Psychiatry.*

**Executive function**	▪ Trail Making Test - B<br>▪ Cambridge Neuropsychological Test Automated Battery - Intradimensional/Extradimensional set shifting<br>▪ Cambridge Neuropsychological Test Automated Battery - Stockings of Cambridge<br>▪ Wisconsin Card Sorting Test categories completed<br>▪ Wisconsin Card Sorting Test perseverative errors<br>▪ Wisconsin Card Sorting Test percentage perseverative errors<br>▪ Wisconsin Card Sorting Test total errors<br>▪ Tower of London	TMT-B<br>CANTAB IED<br><br><br>CANTAB SOC<br><br>WCST categories<br>WCST perseverative<br><br>WCST perseverative<br><br>WCST errors<br>Tower of London

© 2024 Lee M et al. *JAMA Psychiatry.*

eTable 10. Studies that were assumed to overlap

Author	Title	Year	Journal	Overlapping sample with	Sample Size	Country	City	Institution
**An et al.,**	Serum NCAM levels and cognitive deficits in first episode schizophrenia patients versus health controls.	2018	Schizophrenia research	Wu et al., 2016	30 FEP, 30 HC	China	Beijing	Beijing HuiLongGuan Hospital
**Zhang et al.,**	Glucose disturbances, cognitive deficits and white matter abnormalities in first-episode drug-naive schizophrenia.	2020	Molecular psychiatry	Wu et al., 2016	39 FEP, 30 HC	China	Beijing	Beijing HuiLongGuan Hospital
**Xiu et al.,**	Cognitive Deficits and Clinical Symptoms with Hippocampal Subfields in First-Episode and Never-Treated Patients with Schizophrenia.	2021	Cerebral cortex (New York, N.Y. : 1991)	Wu et al., 2016	39 FEP, 30 HC	China	Beijing	Beijing HuiLongGuan Hospital
**Xie et al.,**	Plasma total antioxidant status and cognitive impairments in first-episode drug-naive patients with schizophrenia.	2019	Cognitive neurodynamics	Wu et al., 2016	54 FEP, 50 HC	China	Beijing	Beijing HuiLongGuan Hospital
**Yang M et al., (2)**	Sex-differential associations between cognitive impairments and white matter abnormalities in first episode and drug-naive schizophrenia.	2020	Early intervention in psychiatry	Wu et al., 2016	39 FEP, 30 HC	China	Beijing	Beijing HuiLongGuan Hospital
**Yang M et al., (1)**	Cognitive deficits and white matter abnormalities in never-treated first-episode schizophrenia.	2020	Translational psychiatry	Wu et al., 2016	39 FEP, 30 HC	China	Beijing	Beijing HuiLongGuan Hospital

© 2024 Lee M et al. *JAMA Psychiatry.*

**Qiu, X. et al.,**	The Relationship Between Abnormal Resting-State Functional Connectivity of the Left Superior Frontal Gyrus and Cognitive Impairments in Youth-Onset Drug-Naive Schizophrenia.	2021	Frontiers in psychiatry	Wei et al., 2022	66 FEP, 59 HC	China	Nanjing	Affiliated Brain Hospital of Nanjing Medical University Jiangsu
**Yan et al.,**	Relationships between abnormal neural activities and cognitive impairments in patients with drug-naive first-episode schizophrenia.	2020	BMC psychiatry	Wei et al., 2022	69 FEP, 74 HC	China	Nanjing	Affiliated Brain Hospital of Nanjing Medical University Jiangsu
**Peng et al.,**	Reduced white matter integrity associated with cognitive deficits in patients with drug-naive first-episode schizophrenia revealed by diffusion tensor imaging.	2020	American journal of translational research	Wei et al., 2022	46 FEP, 50 HC	China	Nanjing	Affiliated Brain Hospital of Nanjing Medical University Jiangsu
**Ou et al.,**	Decreased white matter FA values in the left inferior frontal gyrus is a possible intermediate phenotype of schizophrenia: evidences from a novel group strategy.	2018	European archives of psychiatry and clinical neuroscience	Guo et al., 2014	22 FEP, 22 HC	China	Xiangya	Second Xiangya Hospital
**Zong, X et al.,**	DNA Methylation Basis in the Effect of White Matter Integrity Deficits on Cognitive Impairments and Psychopathological Symptoms in Drug-Naive First-Episode Schizophrenia.	2021	Frontiers in psychiatry	Zhao et al., 2022	42 FEP, 38 HC	China	Xinxiang	Second Affiliated Hospital of Xinxiang Medical University

**Zhang et al.,**	Abnormal default-mode network homogeneity and its correlations with neuro-cognitive deficits in drug-naive first-episode adolescent-onset schizophrenia.	2020	Schizophrenia research	Zhao et al., 2022	48 FEP, 31 HC	China	Xinxiang	Second Affiliated Hospital of Xinxiang Medical University
**Duan et al.,**	Reduced Hippocampal Volume and Its Relationship With Verbal Memory and Negative Symptoms in Treatment-Naive First-Episode Adolescent-Onset Schizophrenia.	2021	Schizophrenia bulletin	Zhao et al., 2022	36 FEP, 30 HC	China	Xinxiang	Second Affiliated Hospital of Xinxiang Medical University
**Liu et al.,**	Decreased Resting-State Interhemispheric Functional Connectivity Correlated with Neurocognitive Deficits in Drug-Naive First-Episode Adolescent-Onset Schizophrenia.	2018	The international journal of neuro-psychopharmacology	Zhao et al., 2022	48 FEP, 31 HC	China	Xinxiang	Second Affiliated Hospital of Xinxiang Medical University
**Wang et al.,**	Abnormal long- and short-range functional connectivity in adolescent-onset schizophrenia patients: A resting-state fMRI study.	2018	Progress in neuro-psychopharmacology & biological psychiatry	Zhao et al., 2022	48 FEP, 31 HC	China	Xinxiang	Second Affiliated Hospital of Xinxiang Medical University
**Liu et al.,**	Abnormal neural activity as a potential biomarker for drug-naive first-episode adolescent-onset schizophrenia with coherence regional homogeneity and support vector machine analyses.	2018	Schizophrenia research	Zhao et al., 2022	48 FEP, 31 HC	China	Xinxiang	Second Affiliated Hospital of Xinxiang Medical University

© 2024 Lee M et al. *JAMA Psychiatry.*

**Li X, et al.,**	The effect of serum lipids and short-chain fatty acids on cognitive functioning in drug-naive, first episode schizophrenia patients.	2022	Psychiatry research	Tao et al., 2020	44 FEP, 35 HC	China	Zhengzhou	Zhengzhou University, First affiliated hospital of Zhengzhou

© 2024 Lee M et al. *JAMA Psychiatry.*

eMethods 5. Methods for pooling non-independent cognitive tasks

The data collected for this meta-analysis often included several outcome measures belonging to the same cognitive domain. This is partly due to the MATRICS MCCB, which includes several outcome measures for the domains processing speed and working memory. A meta-analysis assumes that all effect sizes that are included are independent. In order to keep as many effect sizes and outcome measures as possible, we opted to perform three-level meta-analyses, following the example by Harrer et al[8] and Viechtbauer[9] using the metafor[10] package in R. We assumed that effect sizes for cognitive tests (outcome measures) were nested together on one level, to make up a larger cluster (per study) on the higher level. This was expressed in the rma.mv function as: random = ~ 1 | Study/Outcome_measure. We furthermore approximated a variance matrix (V) for the effect sizes within studies using the following formula (example shown for Speed of processing; abbreviated to "sop" in code): V_sop <- vcalc(vi, cluster=Study, obs=Outcome_measure, data=sop_escalc, rho=0.5). Correlation coefficients for within-study correlation of effects were calculated using the individual participant datasets we had access to (our own data from KaSP, data from Olivier et al.[11] and Solis-Vivanco et al.[12]. For the domain Speed of Processing (encompassing the outcome measures TMT-A, BACS-SC and Animal fluency), the within-study correlations ranged between *r* .45 to .70, and the median correlation was .54. For the main meta-analysis a within-study correlation of effects of 0.5 was used. Sensitivity analyses were also conducted using a within-study correlation of 0.3 and 0.7, and yielded identical results as the main analyses.

For the Working Memory domain (encompassing the outcome measures LNS and Spatial Span), we similarly had access to three separate datasets (same as above) of individual participant data and the within-study correlations ranged between *r* .41 to .68, with a median correlation of .56. We used a within-study correlation of effects of 0.5 in the main meta-analysis for working memory as well. Sensitivity analyses were also conducted using a within-study correlation of 0.3 and 0.7, and yielded identical results as the main analyses.

For the domain of Executive Function we only had access to our own individual participant dataset. Correlations between different outcome measures of the WCST yielded an *r* of .69. In our main analysis we opted to use a within-study correlation of 0.6. Sensitivity analyses were also conducted using a within-study correlation of 0.4 and 0.8, and yielded identical results as the main analyses.

© 2024 Lee M et al. *JAMA Psychiatry.*

eMethods 6. Modified Newcastle Ottawa Scale

NEWCASTLE - OTTAWA QUALITY ASSESSMENT SCALE
(adapted for cross sectional studies)

Selection: (Maximum 5 stars)

1) Representativeness of the sample:
   - a) Truly representative of the average in the target population. * (all subjects or random sampling)
   - b) Somewhat representative of the average in the target population. * (non-random sampling)
   - c) Selected group of users.
   - d) No description of the sampling strategy.

2) Sample size:
   - a) Justified and satisfactory. *
   - b) Not justified.

3) Selection of controls:
   - a) Community controls. *
   - b) Convenience sampling, e.g. health care staff, friends and family of researchers
   - c) No description.

4) Ascertainment of the exposure (risk factor):
   - a) Validated measurement tool. **
   - b) Non-validated measurement tool, but the tool is available or described.*
   - c) No description of the measurement tool.

Comparability: (Maximum 2 stars)

1) The subjects in different outcome groups are comparable, based on the study design or analysis. Confounding factors are controlled.
   - a) The study controls for the most important factor (select one). *
   - b) The study control for any additional factor. *

Outcome: (Maximum 2 stars)

1) Assessment of the outcome (cognition):
   - a) Administration of cognitive test was performed in a standardized manner by a trained professional, or a computerized test was used *
   - b) No information on administration or insufficient description

2) Statistical test:
   - a) The statistical test used to analyze the data is clearly described and appropriate, and the measurement of the association is presented, including confidence intervals and the probability level (p value). *
   - b) The statistical test is not appropriate, not described or incomplete.

© 2024 Lee M et al. *JAMA Psychiatry.*

This scale has been adapted from the Newcastle-Ottawa Quality Assessment Scale for cross sectional studies in order to perform a quality assessment of studies for the systematic review, “Cognition in drug-naive first episode psychosis - an updated meta-analysis of neurocognitive function and variability”.

We have not selected one factor that is the most important for comparability, because the variables are not the same in each study. Thus, the principal factor should be identified for each study.

In this scale, the outcome items refers to the measurement and analysis of cognition, even though the articles assessed might have a larger focus on other matters (e.g. biological data or treatment interventions).

© 2024 Lee M et al. *JAMA Psychiatry.*

eMethods 7. Regarding the CVR measure

As mentioned, the coefficient of variation ratio (CVR) is the natural logarithm of the ratio of estimates of population coefficients of variation. A more direct comparison of within-group variability is the log variability ratio (VR), simply defined as the natural logarithm of the ratio of standard deviations for each group. We chose to present the CVR values over the VR values, since we were expecting a large difference in mean values (the previous meta-analysis indicated that controls would outperform the patients). Variance tends to increase as the mean increases[13] and we wanted to remove this effect in our primary results, to give a more unbiased sense of within-group variability differences. We also computed the VR values, and these can be found in eTable 12.

In the CVR analysis, for computational reasons, we could not reverse the test scores where low points equals better performance in the same manner as we did in the meta-analysis of mean differences (where values were multiplied by -1). When comparing the CVR and VR values there was a noticeable difference in the domain of Executive Function between CVR (1.34) and VR (1.71). Given that this domain includes outcome measures where higher scores equals better performance (for e.g. WCST categories completed) and those where higher scores equals worse performance (for e.g. WCST perseverative errors) we decided to perform sensitivity analyses with low and high outcome measures separately (see eTable 13)

When analyzed separately, there appears to be equal within group variability as measured using the CVR in error measures of executive function (CVR = 1.04, no statistically significant difference $p = 0.604$). For the other outcome measures, there is a statistically significant ($p < 0.0001$) greater within-group variability for patients with CVR 2.08.

There is a known ceiling effect in the WCST, particularly for the outcome measure categories completed in normal[14] and gifted[15] subjects, and the discrepancy in CVR values could likely be an artefact of this. We therefore present the combined CVR value for this entire cognitive domain, as this was what was pre-registered in PROSPERO.

Sensitivity analyses were also conducted for two other domains that included outcome measures where low scores equals better performance; processing speed (with TMT A analyzed separately, yielding identical CVR values for TMT-A [CVR = 1.41] and all other speed of processing tests [CVR = 1.42]) and working memory (without CANTAB SWM outcome measures, resulting in CVR = 1.71 compared to a CVR of 1.61 when they were included).

eMethods 8. Regarding Meta-regressions

Meta-regressions were only performed if 10 or more studies reported on the variable in question. A weighted average across the study was used for age as a moderator, and for gender as a moderator the total percentage of females was used.

eDiscussion.

The CVR analysis indicated that patients exhibited greater within-group variability in cognition, compared to the controls. CVR values were consistently higher across cognitive domains, with the highest values in the domains Attention (CVR 1.92) and lowest values in Executive Function (1.34). That patients exhibit so much variability in their performance on tests of attention (almost twice that of controls) might partly be due to the fact that these tasks are very sensitive to sleep deprivation or motivational issues that could arise from the non-medicated

© 2024 Lee M et al. *JAMA Psychiatry.*

state.The large CVR indicates a wide range of performance, so most likely there are also well-preserved attentional capacity in some FEP.

The domain with the lowest CVR was Executive Function. As discussed above in eMethods 7, ceiling effects (for positive outcome measures such as categories completed) in controls makes the combined CVR value more challenging to interpret. Positive outcome measures show a much higher within-group variability for patients using the CVR (2.08), where the mean value is also taken into account (here controls have a higher mean value, but due to the ceiling effect, the variance does not increase with the mean). The VR for positive outcome measures (1.56) (not taking into account the higher mean for controls) still indicates more variability in patients.

For negative outcome measures (errors, or time in TMT B) there is no significant difference when looking at the CVR (1.04). So, when taking into account the greater mean for patients, their variability is about equal to that of controls. If the mean difference is not considered, patients do show considerably more variability (VR 1.80).

In sum, patients perform significantly worse than controls in tests of executive function, both in terms of completing tasks and the amount of errors made (as shown by the meta-analysis of mean values), however (when considering this difference in mean) the within-group variability is primarily higher for patients when it comes to completing tasks and not the amount of errors made.

© 2024 Lee M et al. *JAMA Psychiatry.*

eTable 11. Characteristics of included studies.

Author, year	Country	N FEP	N HC	Age mean (SD)	Gender FEP	Gender HC	Education, mean (SD)	DUP mean (SD)/ Median/Criterion	NOS	Cognitive tests	Included 2014
Yazihan & Yetkin, 2020[16]	Turkey	10	19	FEP: 22.7 (2.4)<br>HC: 22.7 (3.5)	F:0/M:10	F:0/M:19	FEP: 11.23 (2.71)<br>HC: 12.57 (1.91)	NA	5	TMT A, WAIS coding, Animal fluency, FAS fluency, TMT B	No
Goghari, 2013[17]	Canada	19	26	FEP: 18.9 (3.6)<br>HC: 20.9 (2.1)	F:8/M:11	F:9/M:17	FEP: 11.4 (2.7)<br>HC: 14.3 (1.8)	NA	5	CANTAB SWM (between errors)	No
Randau, 2019[18]	Denmark	56	64	FEP: 24.6 (5.8)<br>HC: 24.8 (5.6)	F:25/M:31	F:27/M:37	NA	NA	7	BACS Digit sequence	No
Jessen, 2019[6]	Denmark	105	136	FEP: 24.6 (5.4)<br>HC: 24.3 (5.4)	F:41/M:64	F:57/M:79	NA	NA	8	CANTAB IED (total errors adjusted), CANTAB SOC (problems solved in minimum moves), CANTAB SWM (total errors + strategy)	No
Hong, 2019[19]	China	68	64	FEP: 22.6 (7.7)<br>HC: 23.2 (7.8)	F:39/M:29	F:42/M:22	NA	12.7 (16.6)	6	WCST (categories completed, perseverative errors, total errors, CPT CT3 (correct trials)	No
Li, 2022[20]	China	25	26	FEP: 27.2 (7.1)<br>HC: 30.5 (5.1)	F:10/M:15	F:13/M:13	FEP: 11.9 (3.3)<br>HC: 14.4 (1.5)	>24 (criterion)	4	MCCB	No
Solis Vivanco, 2020[12]	Mexico	63	102	FEP: 25.1 (7.8)<br>HC: 27.4 (11.1)	F:25/M:38	F:45/M:57	FEP: 10.7 (2.9)<br>HC: 14.6 (3.6)	14.6 (18.3)	4	MCCB	No
Olivier, 2015[11]	South Africa	65	101	FEP: 23.8 (6.5)<br>HC: 25.8 (7.3)	F:19/M:46	F:38/M:63	NA	NA	8	MCCB	No
Molina, 2014[21]	Spain	31	23	FEP: 24.9 (5.2)<br>HC: 25.1 (5.0)	F:9/M:22	F:7/M:16	NA	9.96 (22.5)	4	WCST (categories completed, percentage perseverative errors), N-back 2-back (hits)	No
Chang, 2020[22]	Taiwan	51	128	FEP: 28.7 (9.7)<br>HC: 33.3 (11.9)	F:18/M:33	F:68/M:60	FEP: 12.1 (3.2)<br>HC: 14.3 (3.0)	58.1 (87.5)	6	WAIS coding, WAIS digit span	No
Hsu, 2015[23]	Taiwan	30	30	FEP: 28.1 (6.8)<br>HC: 28.3 (7.0)	F:18/M:12	F:18/M:12	FEP: 13.5 (2.1)<br>HC: 13.7 (2.3)	44.4 (64.8)	7	WCST (categories completed, perseverative errors), CPT (d' unmasked)	No

© 2024 Lee M et al. *JAMA Psychiatry.*

Xie, 2021[24]	China	47	43	FEP: 28.5 (6.4)<br>HC: 26.4 (4.9)	F:30/M:17	F:24/M:19	FEP: 14.3 (3.0)<br>HC: 13.4 (2.8)	15.9 (3.8)	7	Animal fluency, WAIS digit span (backwards), BACS Symbol Coding	No
Yang, 2021[25]	China	65	67	FEP: 32.7 (10.6)<br>HC: 34.9 (11.0)	F:27/M:38	F:27/M:40	FEP: 10.4 (3.6)<br>HC: 12.2 (3.4)	21.7 (27.5)	8	HVLT-R, Animal fluency, Fluency actions, TMT A, TMT B, WAIS digit span (backwards)	No
Richard, 2013[4]	USA	50	53	FEP: 23.7 (7.5)<br>HC: 24.8 (7.3)	F:13/M:37	F:26/M:27	FEP: 12.2 (3.0)<br>HC: 14.6 (2.6)	NA	5	AX CPT (d' long delay)	No*
Anhoj, 2018[26]	Denmark	47	47	FEP: 24.6 (NA)<br>HC: 24.7 (NA)	F:18/M:29	F:18/M:29	FEP: 12.1 (2.6)<br>HC: 14.0 (2.7)	14.8 (17.3)	6	BACS Symbol Coding	No
Huang, 2017[27]	China	58	43	FEP: 22.7 (7.6)<br>HC: 23.1 (7.5)	F:29/M:29	F:27/M:16	FEP: 11.4 (2.7)<br>HC: 12.7 (3.8)	15.1 (25.0)	7	CPT-IP, HVLT-R, BVMT-R, Mazes	No
Wei, 2022[28]	China	117	98	FEP: 24.7 (7.0)<br>HC: 26.5 (7.0)	F:31/M:86	F:40/M:58	FEP: 13.18 (2.8)<br>HC: 14.14 (2.3)	15.8 (14.6)	4	CPT-IP, HVLT-R, BVMT-R, Mazes	No
Guo, 2014[29]	China	51	41	FEP: 22.5 (4.1)<br>HC: 22.8 (3.9)	F:18/M:	F:17/M:	FEP: 11.4 (3.3)<br>HC: 11.9 (2.7)	8.4 (6.8)	7	TMT A, BACS Symbol Coding, Animal fluency, Spatial Span, HVLT-R, BVMT-R	No
Wu, 2016[30]	China	79	124	FEP: 25.7 (7.8)<br>HC: 44.7 (8.8)	F:36/M:	F:59/M:	FEP: 12.7 (3.2)<br>HC: 11.8 (3.4)	>60 (criterion)	6	TMT A, BACS Symbol Coding, Animal Fluency, Spatial Span, HVLT-R, BVMT-R, Mazes, CPT-IP, WAIS digit span	No
Guo, 2020[31]	China	57	59	FEP: NA<br>HC: NA	NA	NA	NA		7	CPT-IP, HVLT-R, BMVT-R, Mazes	No
Zhang, 2014[32]	China	163	42	FEP: 25.8 (5.6)<br>HC: 26.9 (4.6)	F:72/M:91	F:22/M:20	FEP: 9.9 (2.0)<br>HC: 10.5 (0.8)	NA	5	N-back 2-back (accuracy)	No
Zhuo, 2013[33]	China	22	23	FEP: 26.6 (7.2)<br>HC: 26.8 (6.5)	F:7/M:15	F:7/M:16	FEP: 12.6 (2.5)<br>HC: 13.6 (2.0)	14.1 (13.8)	7	Spatial Span	No
Yoon, 2014[34]	USA	12	15	FEP: 20.9 (4.2)<br>HC: 21.0 (4.8)	F:2/M:10	F:2/M:13	FEP: 13.1 (2.9)<br>HC: 13.1 (3.0)	NA	8	AX CPT (AX accuracy)	No
Zhao, 2022[35]	China	48	31	FEP: 15.8 (1.6)<br>HC: 15.4 (1.5)	F:27/M:21	F:17/M:14	FEP: 8.9 (2.0)<br>HC: 8.4 (1.6)	5.4 (6.1)	6	TMT A, BACS Symbol Coding, Animal fluency, HVLT-R, BVMT-R, Mazes	No

© 2024 Lee M et al. *JAMA Psychiatry.*

Hu, 2022[36]	China	38	38	FEP: 25.0 (5.0)<br>HC: 24.8 (4.6)	F:13/M:25	F:13/M:25	FEP: 10.5 (2.8)<br>HC: 11.0 (2.9)	NA	6	WCST (categories completed, percentage perseverative errors), TMT B	No
Tao, 2020[37]	China	90	70	FEP: 21.5 (7.7)<br>HC: 23.4 (5.4)	F:46/M:44	F:38/M:32	FEP: 10.4 (2.6)<br>HC: 11.1 (2.4)	5.9 (6.3)	7	CPT-IP, HVLT-R, BVMT-R, Mazes	No
Andersen, 2013[5]	Denmark	48	48	FEP: 25.4 (5.3)<br>HC: 26.6 (5.4)	F:13/M:35	F:13/M:35	FEP: 12.1 (2.7)<br>HC: 14.8 (2.2)	45.5 (55.3)<br>Median: 19.5	7	TMT A, Animal fluency, Fluency FAS, WAIS digit span (backwards), BSRT, WCST (total errors), RCFT	Yes
Andreasen, 1992[38]	USA	13	15	FEP: 34.2 (11.5)<br>HC: 28.8 (6.3)	F:4/M:9	F:6/M:9	FEP: 11.3 (2.0)<br>HC: 16.3 (1.9)	NA	5	Tower of London (nr completed)	Yes
Brickman, 2004[39]	USA	29	17	FEP: 16.1 (2.0)<br>HC: 16.9 (2.4)	F:14/M:15	F:8/M:9	NA	NA	7	TMT A, WAIS digit span, Fluency FAS, SVLT, TMT B	Yes
Buchsbaum, 1992[40]	USA	16	20	FEP: 29.6 (7.2)<br>HC: 27.1 (6.4)	F:0/M:16	F:0/M:20	NA	55.2 (70.8)	4	CPT (d’)	Yes
Chan, 2006[41]	Hong Kong	78	60	FEP: 28.5 (9.8)<br>HC: 27.9 (9.1)	F:29/M:49	F:41/M:19	FEP: 10.8 (2.5)<br>HC: 10.4 (2.1)	8.3 (14.7)	7	TMT A, TMT B, Animal fluency, LNS (longest span), RBANS Figure Recall, WCST (categories completed, perseverative errors), WMS Logical memory	Yes
Fagerlund, 2004[42]	Denmark	25	25	FEP: 27.3 (5.9)<br>HC: NA	NA	NA	NA	Median: 14	6	TMT A, TMT B, Animal fluency, Fluency FAS, CANTAB RVP, CANTAB IED, CANTAB SOC (minimal moves), WCST (categories completed, total errors)	Yes
Finkelstein, 1997[43]	USA	24	44	FEP: 29.0 (8.9)<br>HC: 27.8 (7.0)	F:12/M:12	F:19/M:25	?	42 (46.8)	7	CPT (A’)	Yes
He, 2013[44]	China	80	72	FEP: 25.4 (8.3)<br>HC: 26.6 (8.9)	NA	NA	FEP: 12.1 (3.1)<br>HC: 12.7 (3.5)	9.9 (8.0)	6	TMT A, TMT B, WAIS coding, CANTAB RVP, CANTAB PRM, WMS Logical memory	Yes
Hill, Beers et al., 2004[45]	USA	62	67	FEP: 26.3 (8.9)<br>HC: 28.0 (9.9)	F:26/M:36	F:30/M:37	FEP: 13.3 (2.9)<br>HC: 14.3 (1.8)	Median: 9.4	7	CVLT (total recall)	Yes

© 2024 Lee M et al. *JAMA Psychiatry.*

Hill, Schuepbach et al., 2004[46]	USA	45	33	FEP: 26.1 (8.1)<br>HC: 23.5 (5.3)	F:17/M:28	F:10/M:23	FEP: 13.7 (3.3)<br>HC: 14.9 (1.7)	NA	7	TMT A, TMT B, Fluency FAS, WAIS coding, WAIS digit span, WMS Visual reproduction, WCST (percentage perseverative errors)	Yes
Hilti, 2010[47]	Switz-erland	29	33	FEP: 22.0 (4.0)<br>HC: 23.2 (2.8)	F:5/M:24	F:9/M:24	FEP: 9.5 (1.3)<br>HC: 12.7 (1.5)	NA	7	CANTAB RVP, CANTAB IED, CANTAB SOC (problems solved in minimum moves)	Yes
Hong, 2002[48]	South Korea	17	24	FEP: 29.9 (7.6)<br>HC: 27.6 (5.8)	F:8/M:9	F:13/M:11	FEP: 14.1 (1.7)<br>HC: 14.8 (1.8)	43.2 (44.4)	6	CAT (hits)	Yes
Hu, 2011[49]	China	56	56	FEP: 21.2 (3.4)<br>HC: 21.9 (2.9)	F:19/M:37	F:19/M:37	FEP: 10.9 (1.8)<br>HC: 10.8 (1.5)	10.2 (6.8)	8	TMT A, Animal fluency, Spatial Span (backwards), HVLT-R, BVMT-R, PASAT, WCST (categories completed, perseverative errors)	Yes
Krieger, 2005[50]	Germany	12	12	FEP: 24.6 (5.8)<br>HC: 25.8 (6.2)	F:6/M:6	F:6/M:6	NA	NA	3	N-back 1-back	Yes
Lu, 2012[51]	China	112	63	FEP: 25.2 (4.3)<br>HC: 26.1 (3.7)	F:53/M:59	F:28/M:35	FEP: 12.5 (3.6)<br>HC: 14.8 (2.5)	NA	3	WCST (categories completed, percentage perseverative errors)	Yes
Nejad, 2011[52]	Denmark	23	35	FEP: 26.2 (5.0)<br>HC: 26.8 (5.8)	F:5/M:18	F:11/M:24	NA	NA	8	N-back 2-back (d'), N-back 1-back (d')	Yes
Parellada, 2000[53]	Spain	14	15	FEP: 23.4 (4.3)<br>HC: 22.5 (3.4)	F:14/M:0	F:15/M:0	FEP: 12.6 (3.1)<br>HC: 16.0 (1.5)	15.9 (12.9)	3	WCST (categories completed, total errors)	Yes
Salgado Pineda, 2003[54]	Spain	13	13	FEP: 23.8 (5.7)<br>HC: 23.4 (4.6)	F:0/M:13	F:0/M:13	NA	NA	4	CPT-IP	Yes
van Veelen, 2011[55]	Nether-lands	23	33	FEP: 25.3 (4.6)<br>HC: 24.5 (4.7)	F:0/M:23	F:0/M:33	FEP: 11.2 (2.7)<br>HC: 13.2 (2.4)	4.9 (4.3)	7	Sternberg Working Memory Task	Yes
Wang, 2007[56]	China	112	452	FEP: 22.5 (7.7)<br>HC: 34.0 (11.7)	F:52/M:60	F:231/ M:221	FEP: 11.9 (4.6)<br>HC: 10.3 (3.7)	NA	8	CPT-37 (A')	Yes
Zhang, 2012[57]	China	214	132	FEP: NA<br>HC: NA	F:89/ M:125	F:63/M:69	NA	>60 (criterion)	6	RBANS Immediate memory	Yes

© 2024 Lee M et al. *JAMA Psychiatry.*

Wang, 2018[58]	China	51	52	FEP: 25.5 (7.1)<br>HC: 25.7 (6.4)	NA	NA	FEP: 12.5 (3.0)<br>HC: 12.8 (2.7)	NA	6	TMT A, BACS Symbol Coding, Spatial Span, Animal fluency	No
Lee, 2023	Sweden	42	64	FEP: 26.7 (6.5)<br>HC: 27.1 (5.6)	F:16/M:26	F:33/M:31	FEP: 13.7 (3.2)<br>HC: 15.00 (2.3)	10.1 (14.9)		MCCB<br>WCST (categories completed, perseverative errors)	No
Vyas, 2018[59]	England	20	19	FEP: 31.3 (12.7)<br>HC: 29.2 (9.2)	F:6/M:14	F:7/M:12	NA	NA	7	WCST (categories completed, perseverative errors), CVLT	No

* Richard 2013 includes some overlapping data with Barch 2003, which was part of the 2014 meta-analysis

Mean values for age and education are in years, mean values for DUP (duration of untreated psychosis) is in months. BACS Symbol Coding: Brief Assessment of Cognition in Schizophrenia, Symbol Coding; BSRT: Buschke Selective Reminding Test; BVMT-R: Brief Visuospatial Memory Test-Revised; CANTAB IED: Cambridge Neuropsychological Test Automated Battery intradimensional/extradimensional set shifting; CANTAB PRM: CANTAB Pattern Recognition Memory; CANTAB RVP: CANTAB Rapid Visual Information Processing; CANTAB SOC: CANTAB Stockings of Cambridge; CANTAB SWM: CANTAB Spatial Working Memory; CPT: Continuous Performance Test; CPT-IP: Continuous Performance Test – Identical Pairs; CVLT: California Verbal Learning Test; HVLT-R: Hopkins Verbal Learning Test-Revised; LNS: Letter Number Sequencing; MCCB: MATRICS Consensus Cognitive Battery (includes TMT-A, BACS Symbol Coding, Animal fluency, Spatial Span, LNS, HVLT-R, BVMT-R, Mazes and CPT-IP); PASAT: Paced Auditory Serial Addition Test; RBANS: Repeatable Battery for the Assessment of Neuropsychological Status; RCFT: Rey-Osterrieth Complex Figure Test; SVLT: Serial Verbal Learning Task; TMT-A: Trail Making Test Part A; TMT-B: Trail Making Test Part B; WAIS: Wechsler Adult Intelligence Scale; WMS: Wechsler Memory Scale; WCST: Wisconsin Card Sorting Test

© 2024 Lee M et al. *JAMA Psychiatry.*

eTable 12. Meta-analysis results, mean differences

Cognitive domain	k	NEs	N FEP	N HC	Hedges *g* Effect size	CI 95 %	z	p	Heterogeneity				Publication bias	
									Q	I^2		Prediction interval	Funnel plot assymetry	Egger test
Speed of processing	20	52	1005	1156	-1.16	-1.35, -0.98	-12.33	<0.001	354.0*	66.3[1]	18.3[2]	-2.17, -0.15	N	0.017
Verbal learning	20	20	1347	1297	-1.08	-1.28, -0.88	-10.69	<0.0001	99.0*	81.50		-1.88, -0.27	Y	n.s.
Visual learning	16	16	1002	1028	-1.04	-1.27, -0.82	-9.15	<0.0001	82.7*	81.74		-1.88, -0.21	N	n.s.
Working memory	25	32	1299	1409	-1.04	-1.35, -0.73	-6.60	<0.001	234.6*	26.8[1]	66.7[2]	-2.58, 0.50	Y	0.024
Attention	21	21	1070	1527	-1.03	-1.24, -0.82	-9.53	<0.0001	117.8*	81.59		-1.91, -0.16	N	n.s.
Reasoning/problem solving	10	10	644	718	-0.90	-1.12, -0.68	-7.94	<0.0001	34.2*	72.42		-1.52, -0.27	N	n.s.
Executive function	20	40	938	897	-0.88	-1.07, -0.69	-9.14	<0.001	149.2*	26.3[1]	49.7[2]	-1.67, -0.09	Y	0.049

* Q-statistic significant at p <0.001 level

1. I^2 value for cognitive domain within study (heterogeneity of outcome measures within a study)
2. I^2 value for heterogeneity between studies

© 2024 Lee M et al. *JAMA Psychiatry.*

eTable 13. Meta-analysis results, within-group variability

Cognitive domain	k	NEs	N FEP	N HC	CVR	CI 95 %	z	p	Heterogeneity				VR	CI 95 %	p
									Q	I^2		Prediction interval			
Speed of processing	20	52	1005	1156	1.43	1.27, 1.61	5.94	<0.001	223.10*	33.2[1]	43.9[2]	0.81, 2.55	1.25	1.10, 1.42	<0.001
Verbal learning	20	20	1347	1297	1.55	1.40, 1.72	8.23	<0.0001	55.67*	66.67		1.05, 2.29	1.20	1.10, 1.33	<0.0001
Visual learning	16	16	1002	1028	1.87	1.63, 2.15	8.99	<0.0001	82.70*	60.51		1.15, 3.05	1.44	1.28, 1.62	<0.0001
Working memory	25	32	1299	1409	1.61	1.37, 1.90	5.72	<0.001	206.53*	21.1[1]	66.2[2]	0.74, 3.54	1.35	1.21, 1.50	<0.001
Attention	21	21	1070	1527	1.92	1.62, 2.27	7.57	<0.0001	97.01*	85.77		0.94, 3.91	1.56	1.31, 1.86	<0.0001
Reasoning/problem solving	10	10	644	718	1.46	1.31, 1.64	6.60	<0.0001	15.22	43.65		1.13, 1.89	1.16	1.04, 1.30	0.0205
Executive function	20	40	938	897	1.34	1.30, 1.58	3.38	0.0007	280.98*	86.48		0.50, 3.57	1.71	1.48, 1.98	<.0001

* Q-statistic significant at p <0.001 level

1. I^2 value for cognitive domain within study (heterogeneity of outcome measures within a study)

2. I^2 value for heterogeneity between studies

© 2024 Lee M et al. *JAMA Psychiatry.*

eTable 14. Meta-analysis results, within-group variability (sensitivity analysis with separate analysis for negative outcome measures)

Cognitive domain	k	NEs	N FEP	N HC	CVR	CI 95 %	z	p	Heterogeneity				VR	CI 95 %	p
									Q	I^2		Prediction interval			
Speed of processing	20	37	1005	1156	1.42	1.26, 1.60	5.70	<0.001	175.10*	47.4[1]	31.0[2]	0.80, 2.53	1.02	0.91, 1.15	<0.001
Speed of processing – TMT A	15	15	718	712	1.41	1.20, 1.66	4.08	<0.0001	47.44*	70.99		0.81, 2.45	2.13	1.75, 2.59	<0.001
Verbal learning	20	20	1347	1297	1.55	1.40, 1.72	8.23	<0.0001	55.67*	66.67		1.05, 2.29	1.20	1.10, 1.33	<0.0001
Visual learning	16	16	1002	1028	1.87	1.63, 2.15	8.99	<0.0001	82.70*	60.51		1.15, 3.05	1.44	1.28, 1.62	<0.0001
Working memory[3]	23	29	1175	1247	1.71	1.47, 1.99	6.90	<0.001	159.48*	29.7[1]	54.4[2]	0.85, 3.43	1.35	1.20, 1.52	<0.001
Attention	21	21	1070	1527	1.92	1.62, 2.27	7.57	<0.0001	97.01*	85.77		0.94, 3.91	1.56	1.31, 1.86	<0.0001
Reasoning/problem solving	10	10	644	718	1.46	1.31, 1.64	6.60	<0.0001	15.22	43.65		1.13, 1.89	1.16	1.04, 1.30	0.0205
Executive function[4]	14	14	661	641	2.08	1.60, 2.70	5.48	<0.0001	85.99*	86.48		0.83, 5.24	1.56	1.23, 1.97	0.0002
Executive function – Error measures[5]	19	26	925	882	1.04	0.89, 1.22	0.52	0.604	110.41*	75.8[1]	0[2]	0.52, 2.10	1.80	1.50, 2.16	<0.001

* Q-statistic significant at p <0.001 level

1. I^2 value for cognitive domain within study (heterogeneity of outcome measures within a study)

2. I^2 value for heterogeneity between studies

3. Not including CANTAB SWM measures

4. WCST categories completed, CANTAB SOC probability of minimum moves, Tower of London nr completed

5. WCST total errors, WCST perseverative errors, WCST percentage perseverative errors, CANTAB IED errors adjusted, CANTAB SOC mean moves, TMT B seconds

© 2024 Lee M et al. *JAMA Psychiatry.*

eTable 15. Meta-regressions

	k	nEs	B	SE	z	p	95 % CI	R2
**Verbal memory**								
Age	18	18	0.017	0.0208	0.81	0.418	-0.024, 0.058	0.00%
Perc female	18	18	-0.215	1.3700	-0.90	0.875	-2.900, 2.470	0.00%
Years of education	15	15	0.080	0.0769	1.03	0.302	-0.071, 0.230	0.32%
Publication year	20	20	-0.016	0.0174	-0.84	0.366	-0.050, 0.018	1.83%
NOS	19	19	-0.002	0.0887	-0.02	0.984	-0.176, 0.172	0.00%
**Visual memory**								
Age	15	15	0.029	0.0257	1.11	0.267	-0.022, 0.079	1.20%
Perc female	14	14	-0.479	1.3113	-0.37	0.715	-3.049, 2.091	0.00%
Years of education	14	14	0.061	0.0829	0.74	0.462	-0.102, 0.223	0.00%
Publication year	16	16	-0.027	0.0201	-1.35	0.177	-0.067, 0.012	7.76%
NOS	15	15	0.070	0.0956	0.73	0.462	-0.117, 0.258	0.00%
**Mazes/Problem-solving**								
Age	9	9	-	-	-	-	-	-
Perc female	9	9	-	-	-	-	-	-
Years of education	8	8	-	-	-	-	-	-
Publication year	10	10	0.026	0.0440	0.59	0.555	-0.060, 0.112	0.00%
NOS	9	9	-	-	-	-	-	-
**Speed of processing**								
Age	20	52	-0.010	0.019	-0.49	0.621	-0.047, 0.028	-
Perc female	17	44	0.444	0.863	0.51	0.607	-1.247, 2.135	-
Years of education	17	44	-0.083	0.074	-1.12	0.264	-0.227, 0.062	-
Publication year	20	52	-0.024	0.015	-1.59	0.112	-0.053, 0.006	-
NOS	19	49	0.112	0.082	1.37	0.171	-0.048, 0.272	-
**Working memory**								
Age	25	32	0.045	0.038	1.17	0.243	-0.030, 0.120	-
Perc female	24	31	3.127	1.573	1.99	0.047*	0.044, 6.211	-
Years of education	18	22	0.116	0.169	0.69	0.490	-0.214, 0.447	-
Publication year	25	32	0.043	0.029	1.50	0.134	-0.013, 0.100	-
NOS	24	30	0.101	0.114	0.88	0.379	-0.123, 0.325	-
**Attention**								
Age	20	20	-0.004	0.0312	0.89	0.891	-0.065, 0.057	0.00%
Perc female	17	17	-0.343	0.7966	-0.43	0.667	-1.904, 1.218	0.00%
Years of education	14	14	0.033	0.1105	0.30	0.767	-0.184, 0.249	0.00%
Publication year	21	21	-0.021	0.0126	-1.64	0.102	-0.046, 0.004	12.55%
NOS	20	20	0.142	0.0786	1.81	0.070	-0.012, 0.296	14.55%
**Executive function**								
Age	20	40	-0.019	0.028	-0.66	0.507	-0.075, 0.037	-
Perc female	18	34	0.318	0.630	0.51	0.613	-0.916, 1.552	-
Years of education	14	25	-0.021	0.086	-0.24	0.810	-0.189, 0.148	-
Publication year	20	40	-0.002	0.013	-0.12	0.905	-0.028, 0.025	-
NOS	19	38	0.088	0.066	1.33	0.184	-0.042, 0.218	-

© 2024 Lee M et al. *JAMA Psychiatry.*

eFigure 1. Forest plot for Processing Speed, mean differences

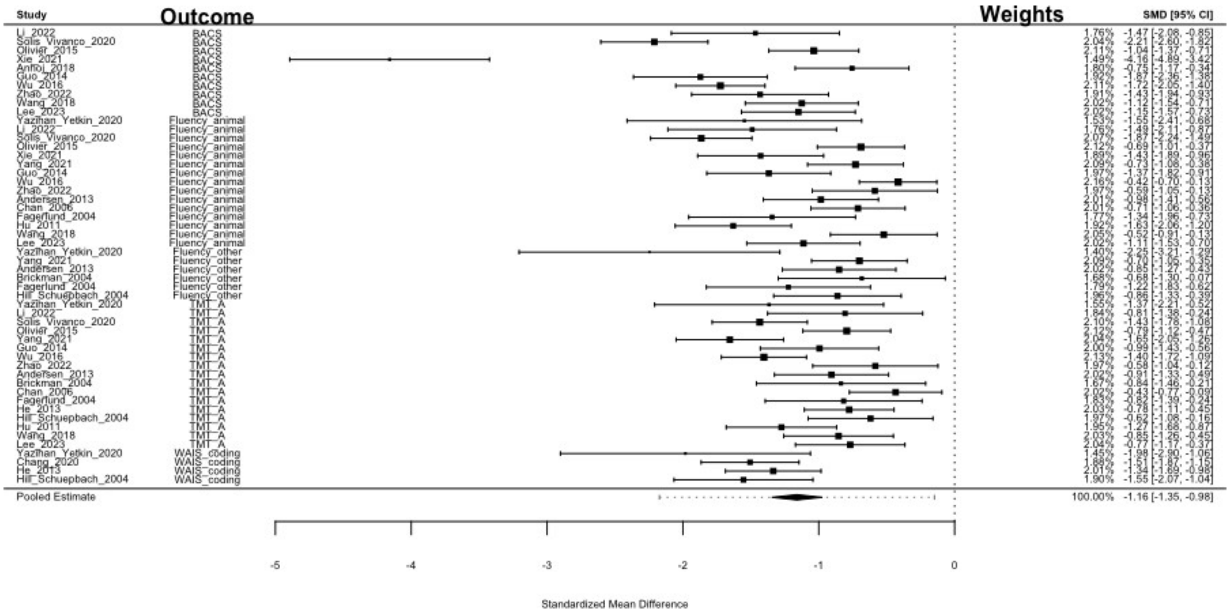

eFigure 2. Forest plot for Verbal learning, mean differences

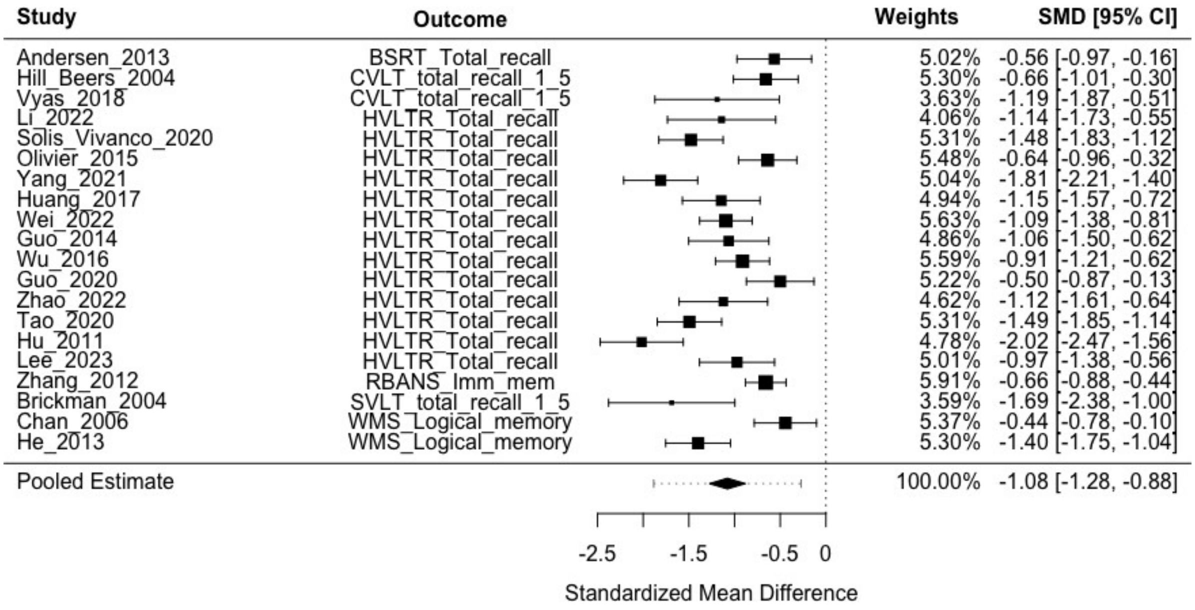

© 2024 Lee M et al. *JAMA Psychiatry.*

eFigure 3. Forest plot for Visual Learning, mean differences

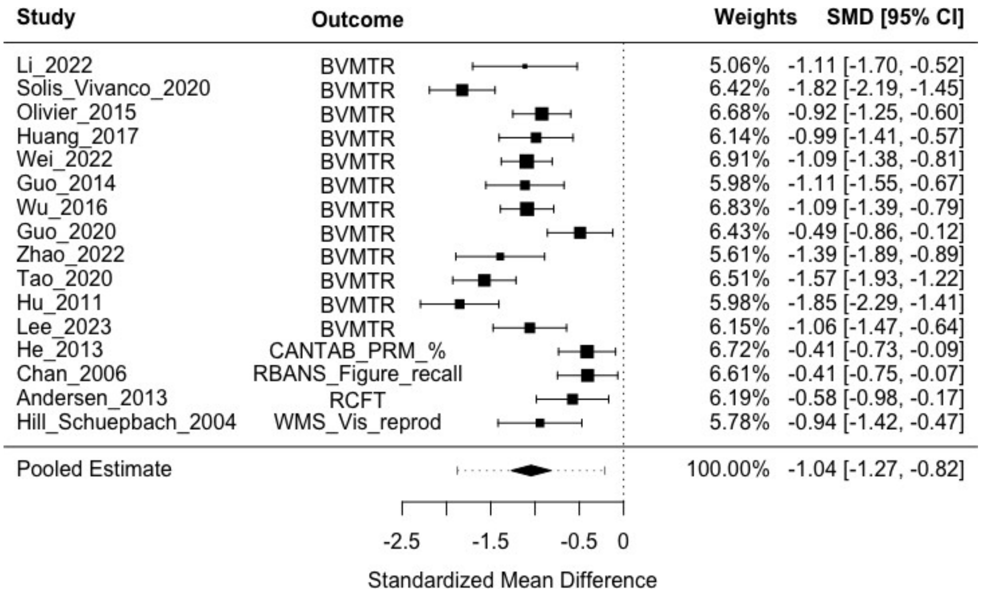

eFigure 4. Forest plot for Working Memory, mean differences

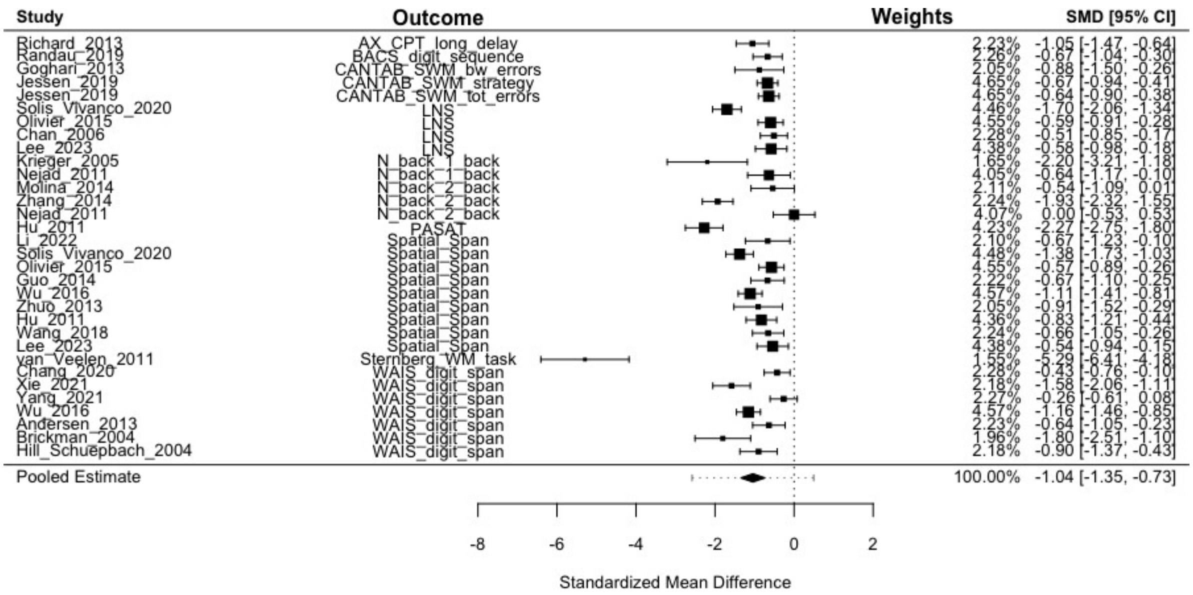

© 2024 Lee M et al. *JAMA Psychiatry.*

eFigure 5. Forest plot for Attention, mean differences

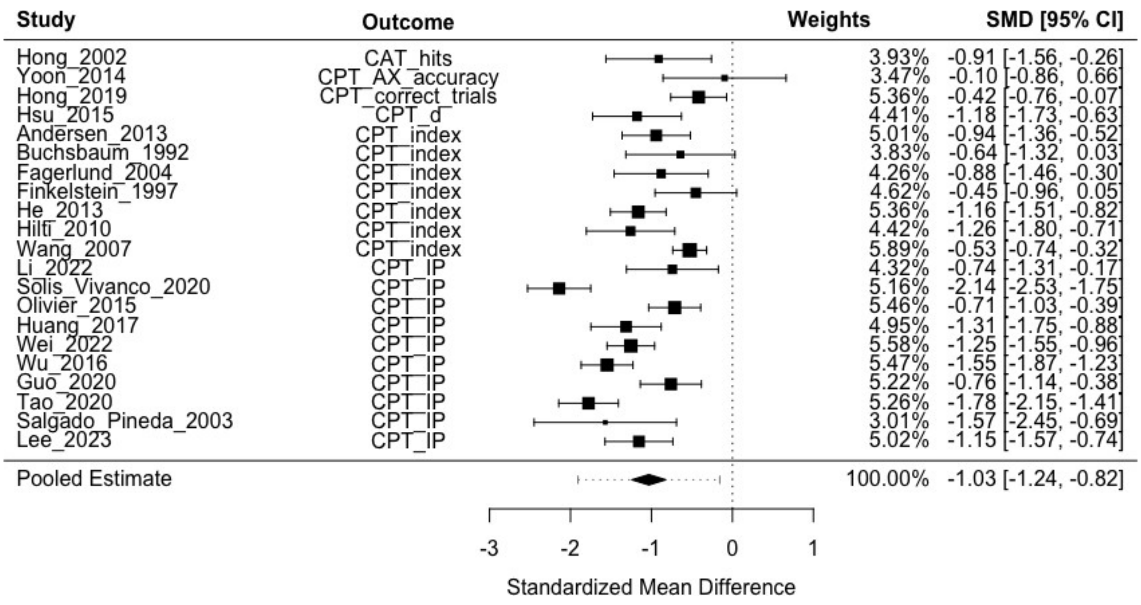

eFigure 6. Forest plot for Reasoning/Problem-solving, mean differences

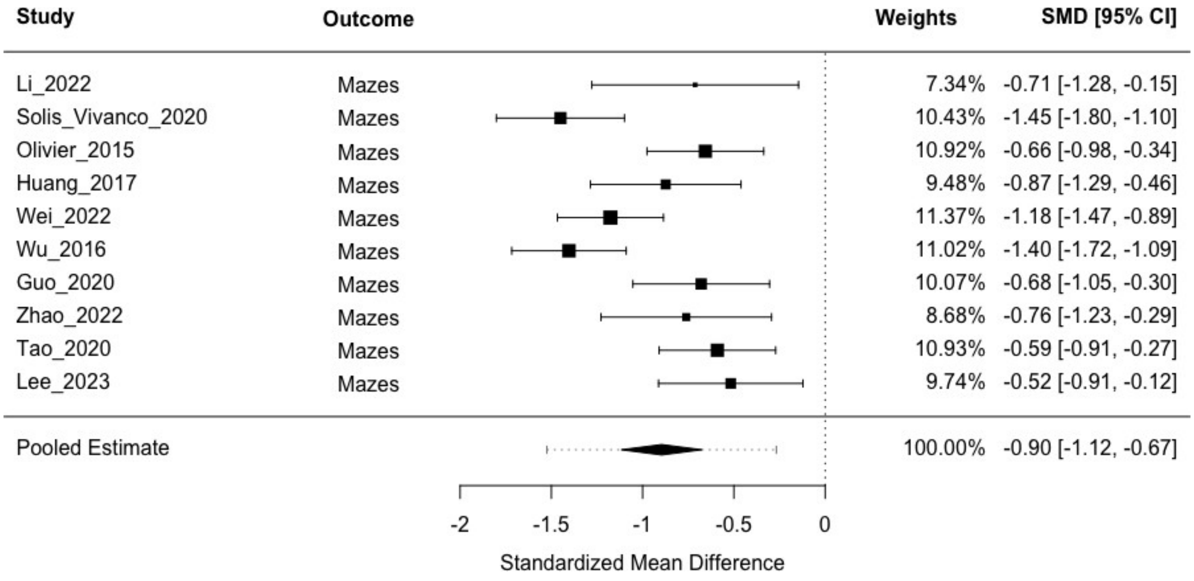

© 2024 Lee M et al. *JAMA Psychiatry.*

eFigure 7. Forest plot for Executive Function, mean differences

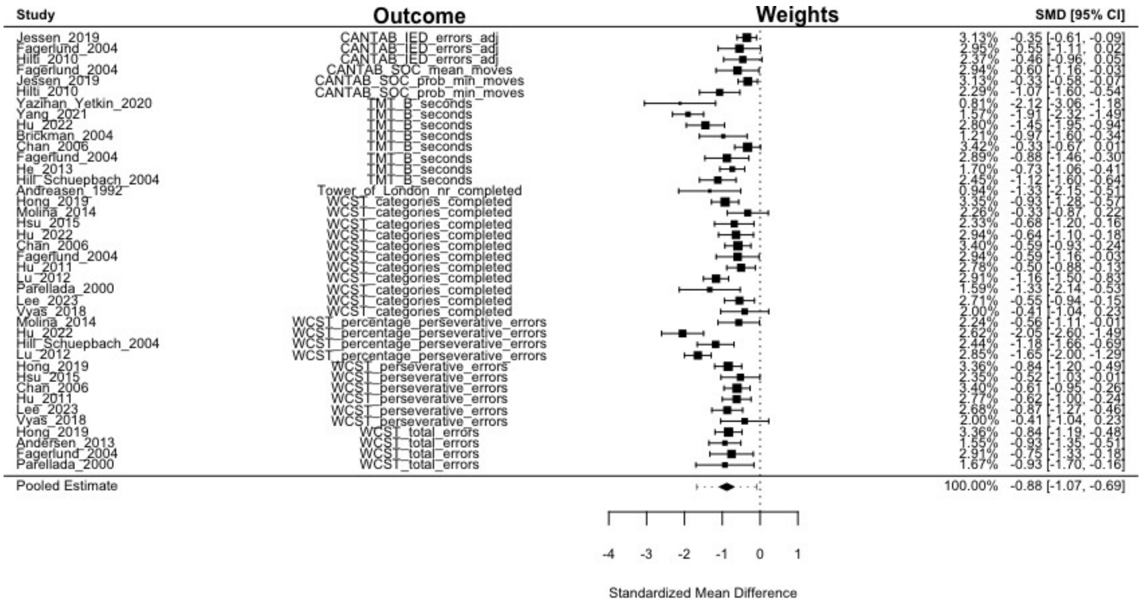

eFigure 8. Forest plot for Processing Speed, CVR variability

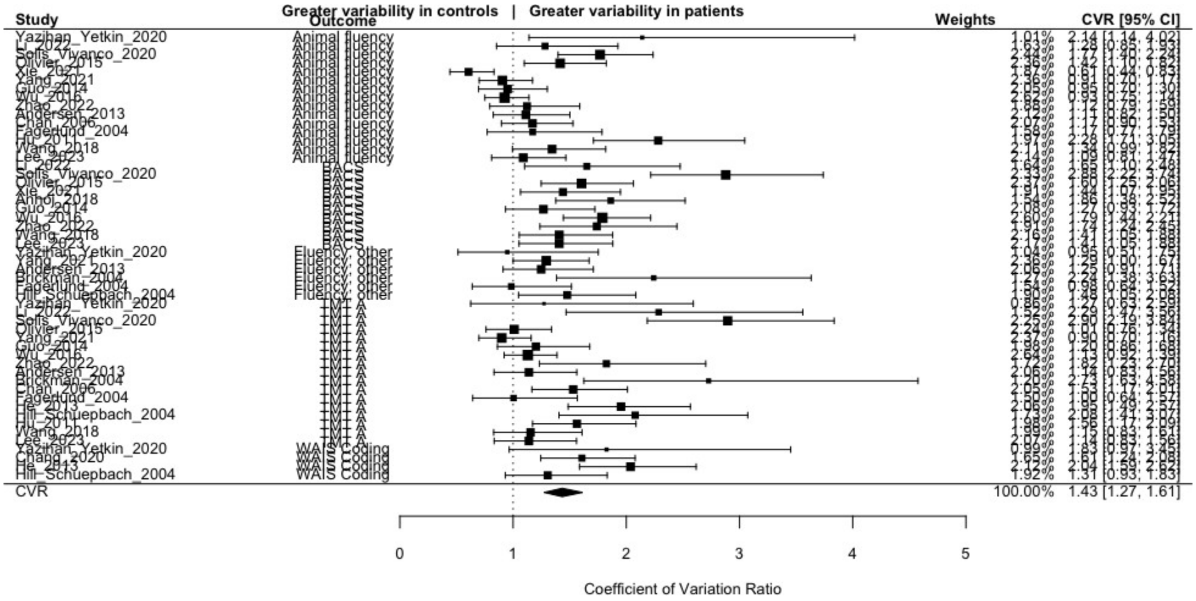

© 2024 Lee M et al. *JAMA Psychiatry.*

eFigure 9. Forest plot for Verbal Learning, CVR variability

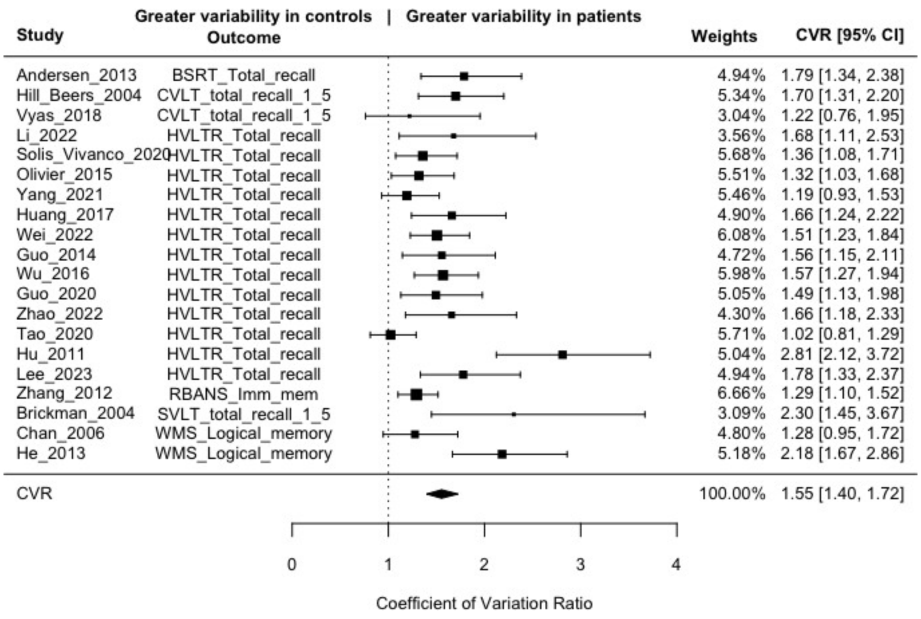

eFigure 10. Forest plot for Visual Learning, CVR variability

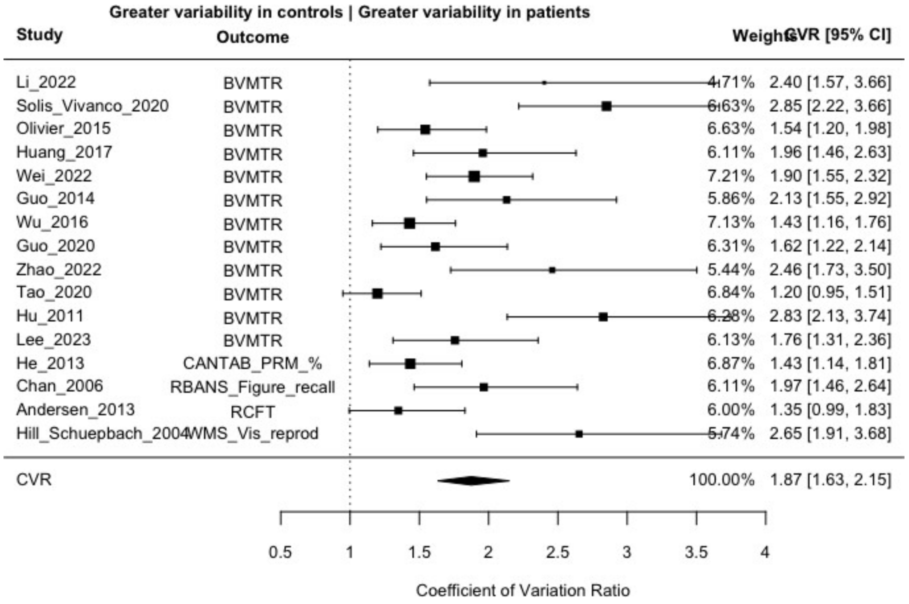

© 2024 Lee M et al. *JAMA Psychiatry.*

eFigure 11. Forest plot for Working Memory, CVR variability

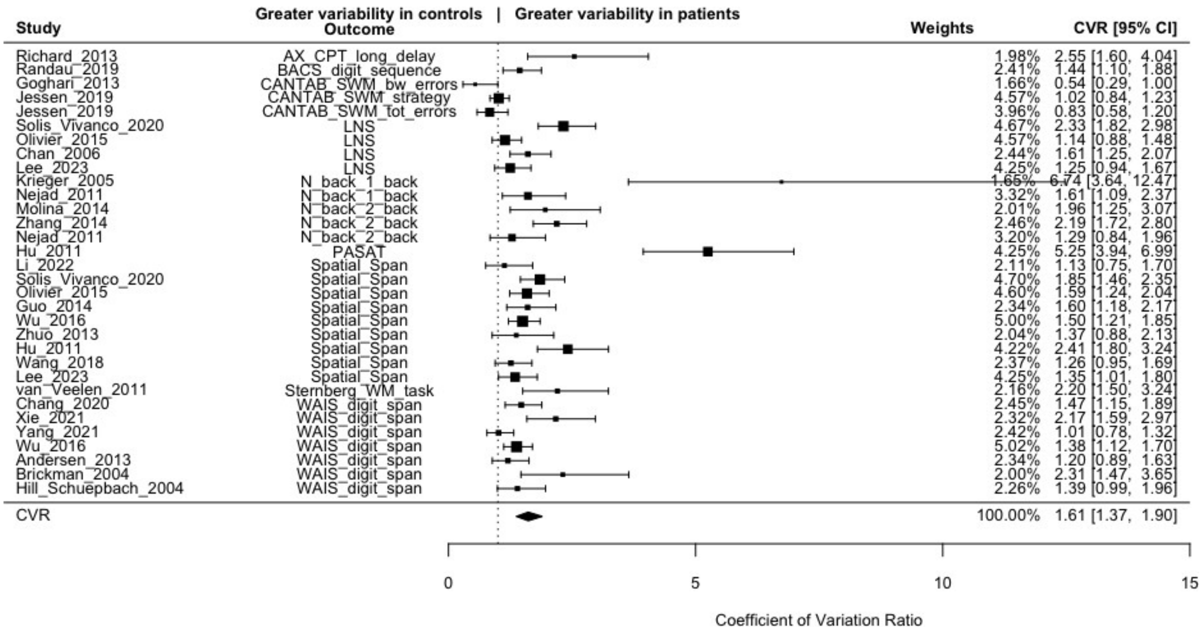

eFigure 12. Forest plot for Attention, CVR variability

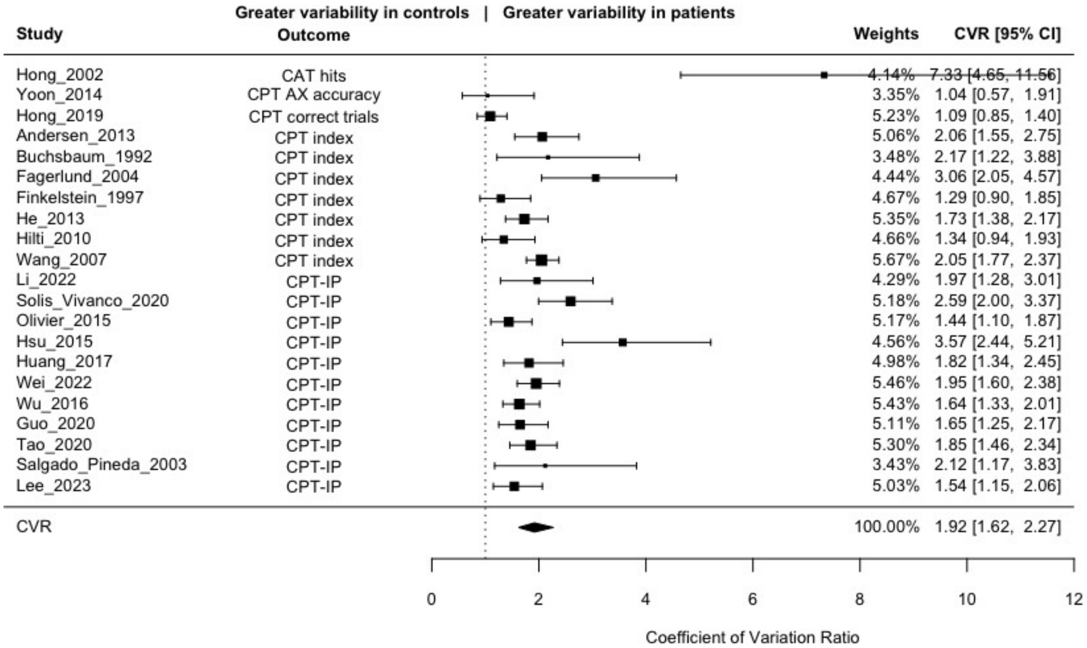

© 2024 Lee M et al. *JAMA Psychiatry.*

eFigure 13. Forest plot for Reasoning/Problem-solving, CVR variability

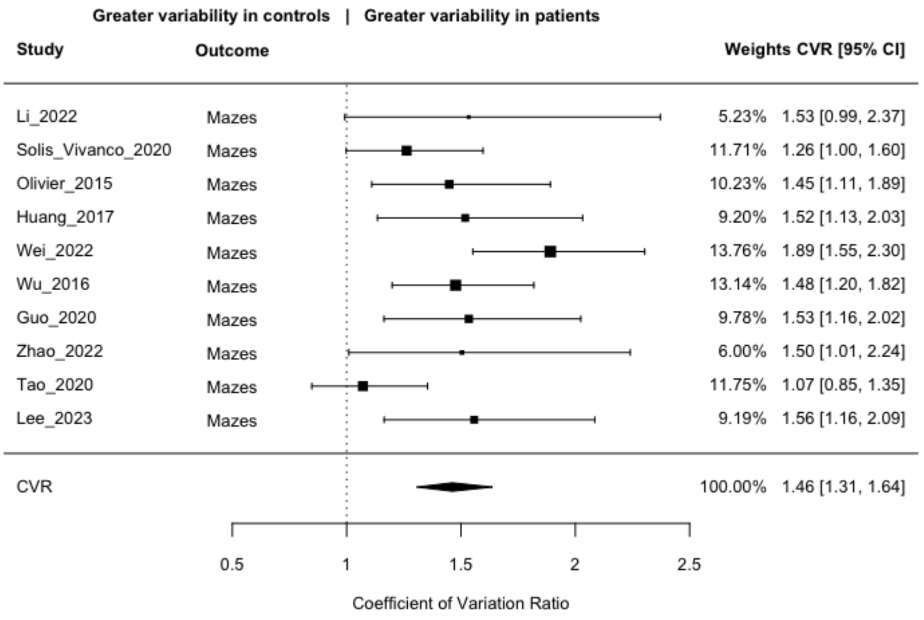

eFigure 14. Forest plot for Executive Function, CVR variability

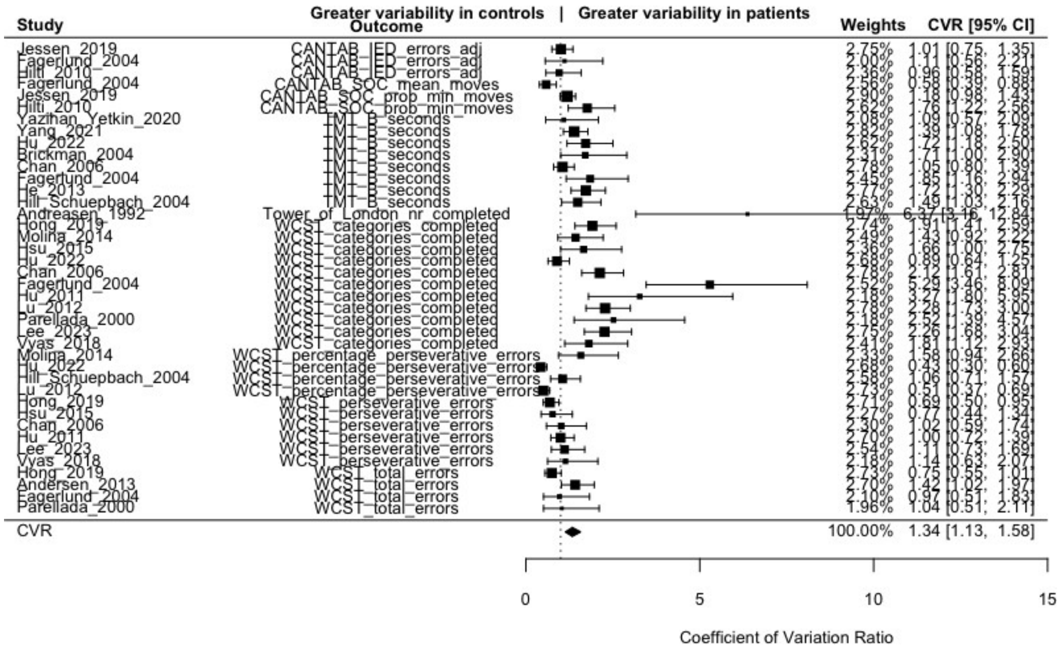

© 2024 Lee M et al. *JAMA Psychiatry.*

eFigure 15. Funnel plot for Processing Speed

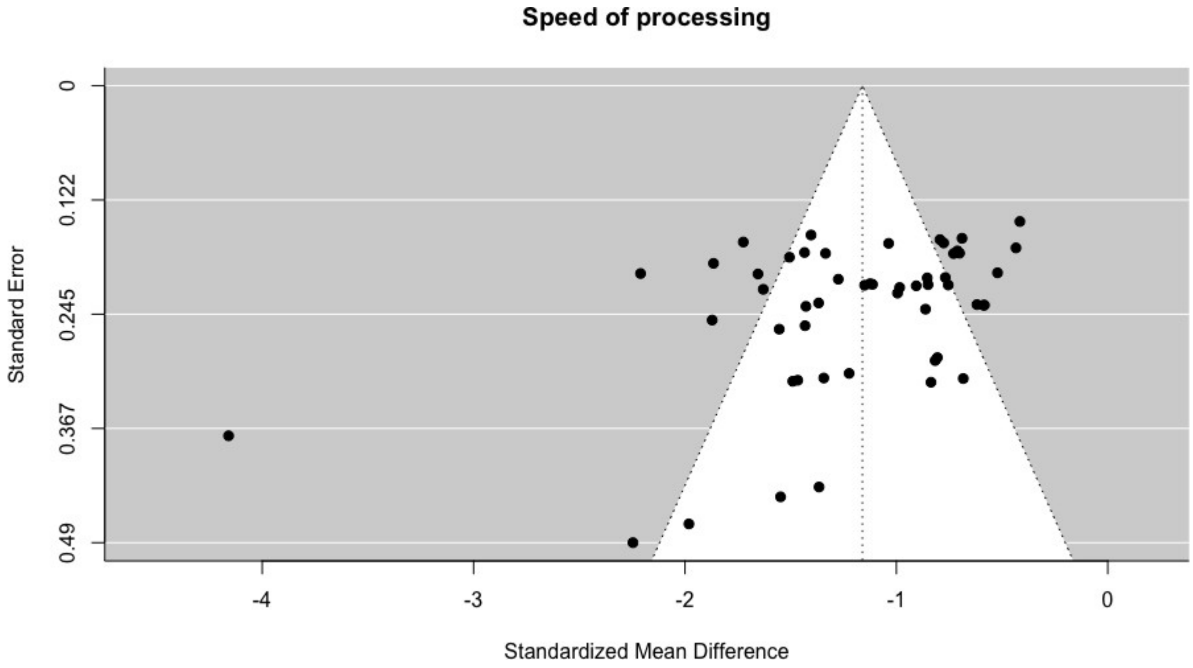

eFigure 16. Funnel plot for Verbal Learning

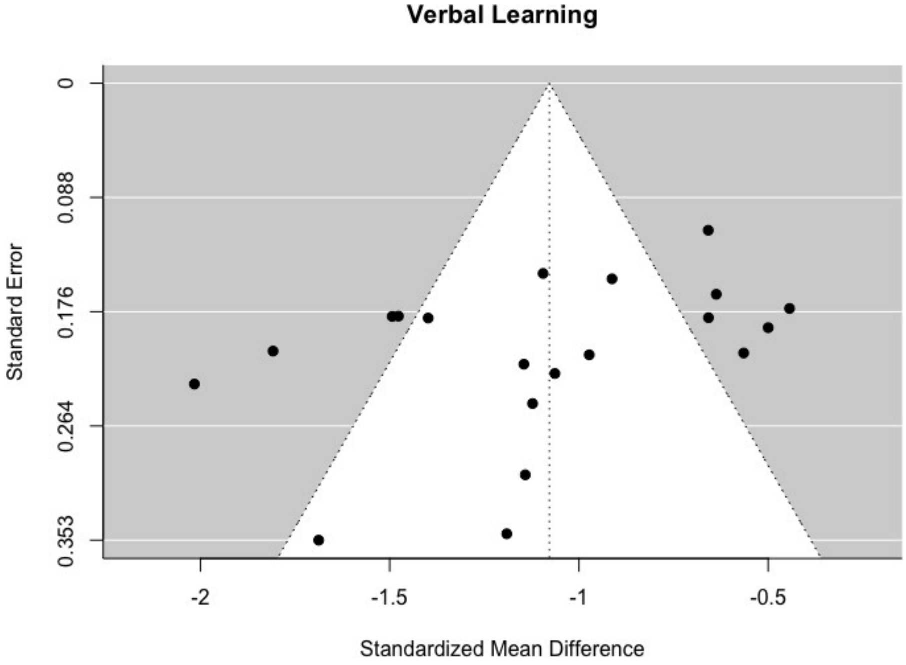

© 2024 Lee M et al. *JAMA Psychiatry.*

eFigure 17. Funnel-plot for Visual Learning

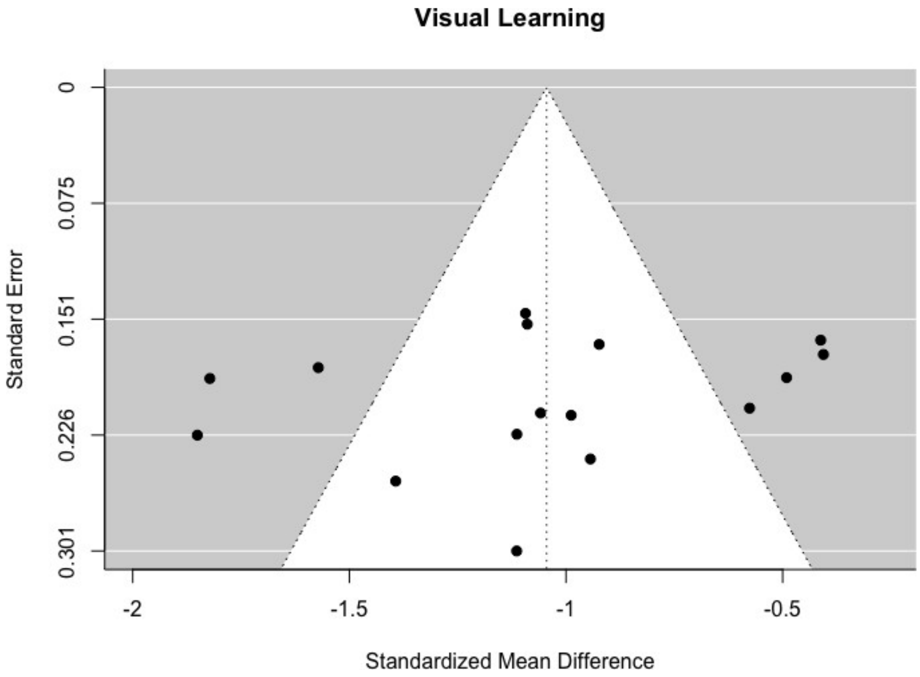

eFigure 18. Funnel-plot for Working Memory

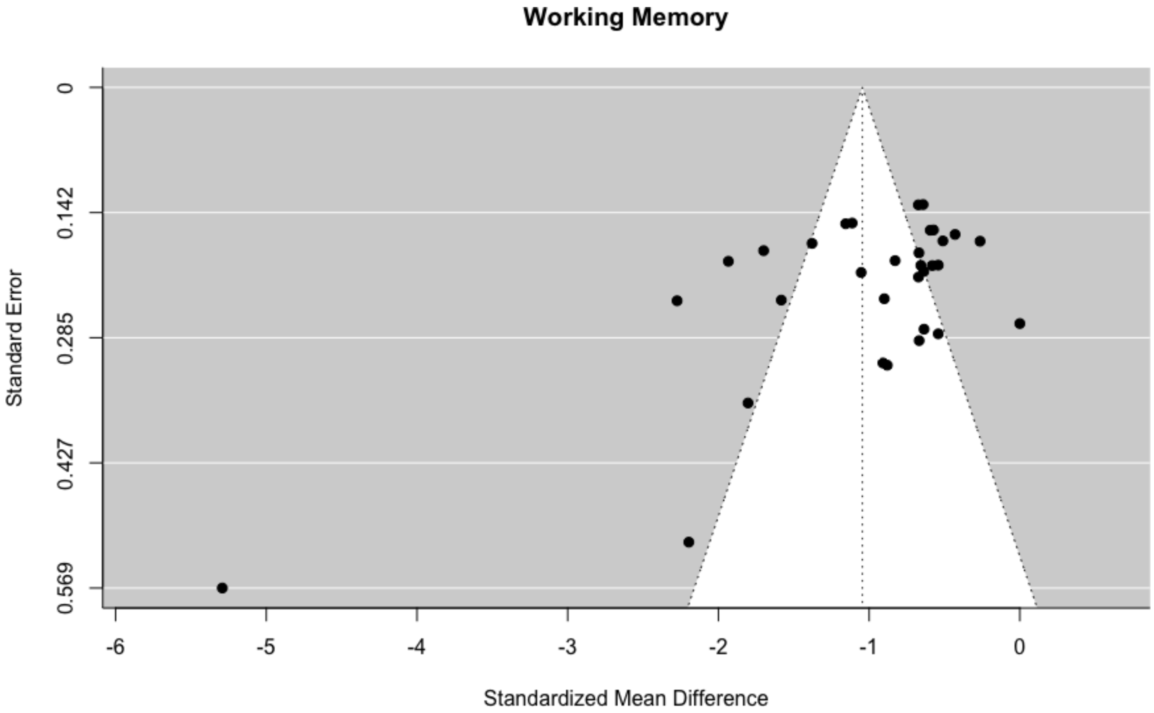

© 2024 Lee M et al. *JAMA Psychiatry.*

eFigure 19. Funnel-plot for Attention

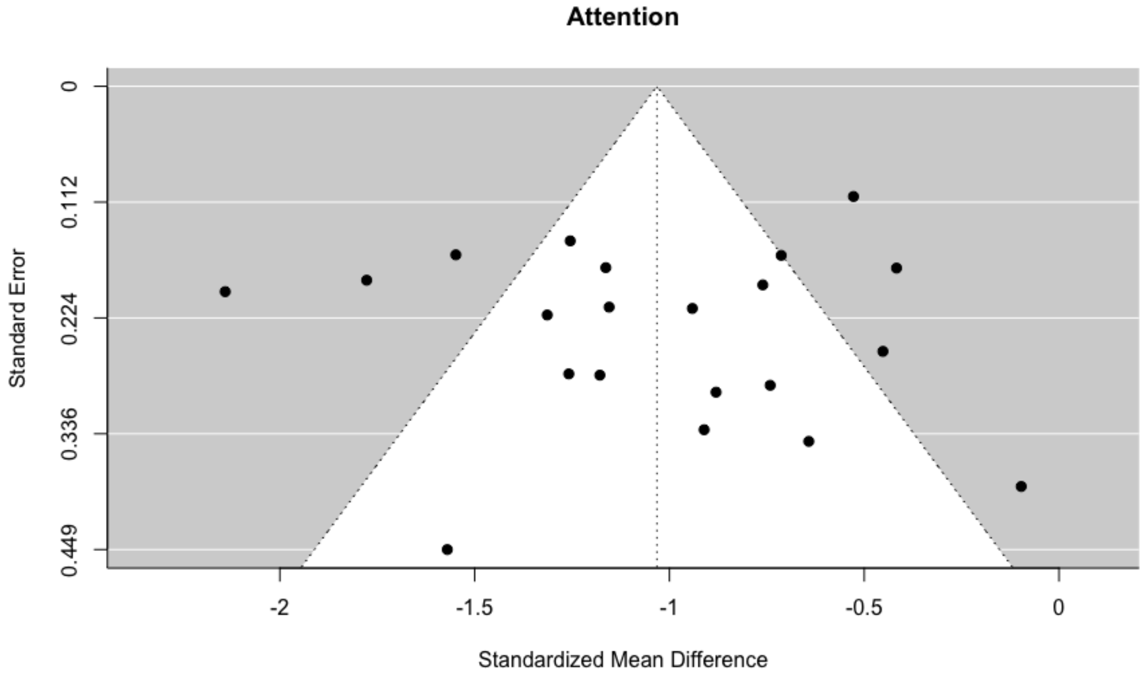

eFigure 20. Funnel-plot for Reasoning/Problem-solving

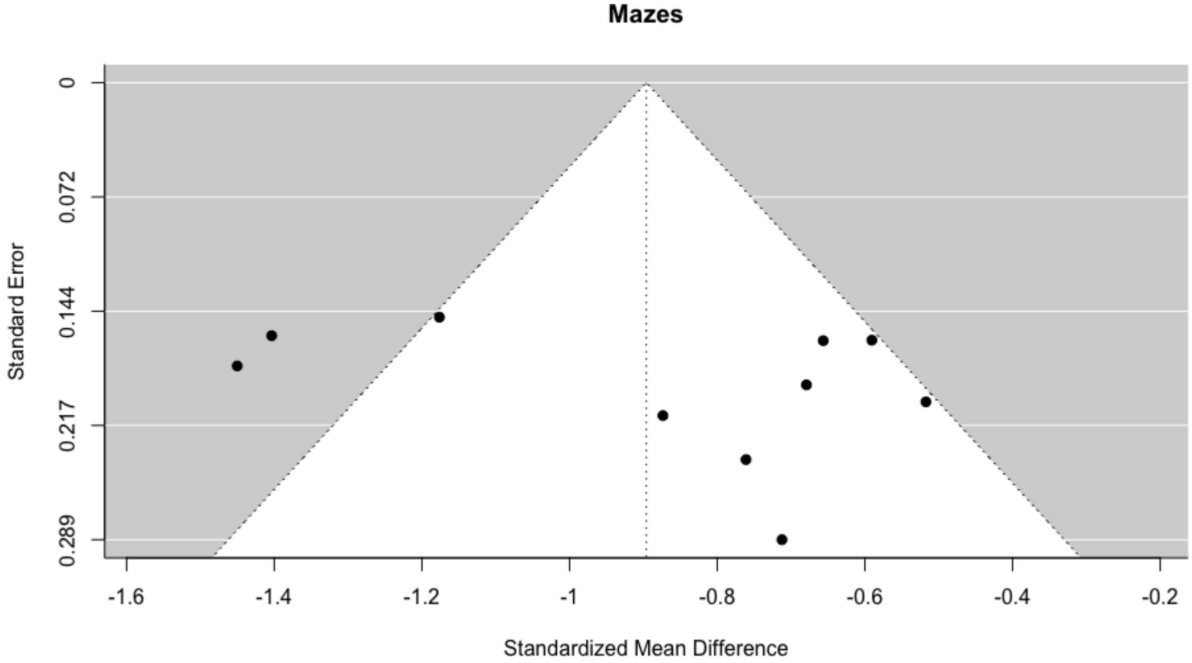

© 2024 Lee M et al. *JAMA Psychiatry.*

eFigure 21. Funnel-plot for Executive Function

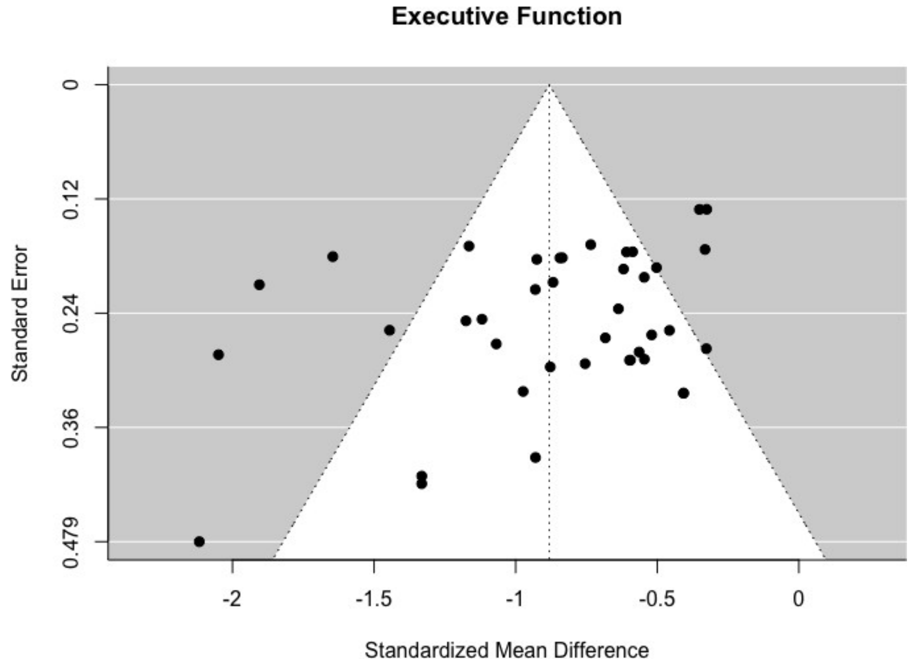

© 2024 Lee M et al. *JAMA Psychiatry.*

References

1. Fatouros-Bergman H, Cervenka S, Flyckt L, Edman G, Farde L. Meta-analysis of cognitive performance in drug-naive patients with schizophrenia. *Schizophr Res*. 2014. doi:10.1016/j.schres.2014.06.034

2. McCreadie RG, Latha S, Thara R, Padmavathi R, Ayankaran JR. Poor memory, negative symptoms and abnormal movements in never-treated Indian patients with schizophrenia. *Br J Psychiatry*. 1997;171:360-363.

3. Barch DM, Braver TS, Carter CS, MacDonald AW, Cohen JD. Context-processing deficits in schizophrenia: Diagnostic specificity, 4-week course, and relationships to clinical symptoms. *J Abnorm Psychol*. 2003;112(1):132-143. doi:10.1037/0021-843X.112.1.132

4. Richard AE, Carter CS, Cohen JD, Cho RY. Persistence, diagnostic specificity and genetic liability for context-processing deficits in schizophrenia. *Schizophr Res*. 2013;147(1):75-80. doi:10.1016/j.schres.2013.02.020

5. Andersen R, Fagerlund B, Rasmussen H, et al. The influence of impaired processing speed on cognition in first-episode antipsychotic-naïve schizophrenic patients. *Eur Psychiatry*. 2013;28(6):332-339. doi:10.1016/j.eurpsy.2012.06.003

6. Jessen K, Mandl RCW, Fagerlund B, et al. Patterns of Cortical Structures and Cognition in Antipsychotic-Naïve Patients With First-Episode Schizophrenia: A Partial Least Squares Correlation Analysis. *Biol Psychiatry Cogn Neurosci Neuroimaging*. 2019;4(5):444-453. doi:10.1016/j.bpsc.2018.09.006

7. Derringer J (University of I. A simple correction for non independent tests. *PsyArxiv*. 2018;15(29):7577-7588. https://www.uam.es/gruposinv/meva/publicaciones jesus/capitulos_espanyol_jesus/2005_motivacion para el aprendizaje Perspectiva alumnos.pdf%0Ahttps://www.researchgate.net/profile/Juan_Aparicio7/publication/253571379_Los_estudios_sobre_el_cambio_conceptual_.

8. Harrer M, Cuijpers P, Furukawa TA, Ebert DD. Doing Meta-Analysis with R: A Hands-On Guide. In: Boca Raton, FL and London: Chapman & Hall/CRC Press; 2021. https://bookdown.org/MathiasHarrer/Doing_Meta_Analysis_in_R/multilevel-ma.html.

9. Viechtbauer W. The metafor Package, Meta-Analysis Package for R - Tips: Forest Plot with Aggregated Values. https://www.metafor-project.org/doku.php/tips:forest_plot_with_aggregated_values#fn__1. Accessed August 10, 2023.

10. Viechtbauer W. Conducting meta-analyses in R with the metafor package. *J Stat Softw*. 2010;36(3):1-48.

11. Olivier MR, Killian S, Chiliza B, et al. Cognitive performance during the first year of treatment in first-episode schizophrenia: A case-control study. *Psychol Med*. 2015;45(13):2873-2883. doi:10.1017/S0033291715000860

12. Solís-Vivanco R, Rangel-Hassey F, León-Ortiz P, Mondragón-Maya A, Reyes-Madrigal F, De La Fuente-Sandoval C. Cognitive Impairment in Never-Medicated Individuals on the Schizophrenia

© 2024 Lee M et al. *JAMA Psychiatry.*

Spectrum. *JAMA Psychiatry*. 2020;77(5):543-545. doi:10.1001/jamapsychiatry.2020.0001

13. Eisler Z, Bartos I, Kertész J. Fluctuation scalin in complex systems: Taylor's law and beyond. *Adv Phys*. 2008;57(1):89-142.

14. Paolo AM, Axelrod BN, Tröster AI. Test-retest stability of the Wisconsin Card Sorting Test. *Assessment*. 1996;3(2):137-143. doi:10.1177/107319119600300205

15. Arffa S, Lovell M, Podell K, Goldberg E. Wisconsin Card Sorting Test performance in above average and superior school children: Relationship to intelligence and age. *Arch Clin Neuropsychol*. 1998;13(8):713-720. doi:10.1016/S0887-6177(98)00007-9

16. Yazihan NT, Yetkin S. Sleep, sleep spindles, and cognitive functions in drug-naive patients with first-episode psychosis. *J Clin Sleep Med*. 2020;16(12):2079-2087. doi:10.5664/jcsm.8776

17. Goghari VM, Smith GN, Honer WG, et al. Effects of eight weeks of atypical antipsychotic treatment on middle frontal thickness in drug-naïve first-episode psychosis patients. *Schizophr Res*. 2013;149(1-3):149-155. doi:10.1016/j.schres.2013.06.025

18. Randau M, Oranje B, Miyakoshi M, et al. Attenuated mismatch negativity in patients with first-episode antipsychotic-naive schizophrenia using a source-resolved method. *NeuroImage Clin*. 2019;22(February). doi:10.1016/j.nicl.2019.101760

19. Hong W, Zhao Z, Shen Z, et al. Uncoupled relationship in the brain between regional homogeneity and attention function in first-episode, drug-naïve schizophrenia. *Psychiatry Res - Neuroimaging*. 2019;294(June). doi:10.1016/j.pscychresns.2019.110990

20. Li D, Zhang X, Kong Y, et al. Lack of neural load modulation explains attention and working memory deficits in first-episode schizophrenia. *Clin Neurophysiol*. 2022;136:206-218. doi:10.1016/j.clinph.2022.02.001

21. Molina V, Taboada D, Aragüés M, Hernández JA, Sanz-Fuentenebro J. Greater clinical and cognitive improvement with clozapine and risperidone associated with a thinner cortex at baseline in first-episode schizophrenia. *Schizophr Res*. 2014;158(1-3):223-229. doi:10.1016/j.schres.2014.06.042

22. Chang WH, Chen KC, Tseng HH, et al. Bridging the associations between dopamine, brain volumetric variation and IQ in drug-naïve schizophrenia. *Schizophr Res*. 2020;220:248-253. doi:10.1016/j.schres.2020.03.005

23. Hsu SE, Chin Chen K, Lee LT, et al. Comparison of cognitive deficits among drug-naive patients with schizophrenia and major depressive disorder. *J Affect Disord*. 2015;175:133-138. doi:10.1016/j.jad.2014.12.059

24. Xie YJ, Xi Y Bin, Cui LB, et al. Functional connectivity of cerebellar dentate nucleus and cognitive impairments in patients with drug-naive and first-episode schizophrenia. *Psychiatry Res*. 2021;300(127). doi:10.1016/j.psychres.2021.113937

25. Yang H, Xiao W, Yang M, Wang Y, Zhang X. Decreased neuregulin1β1 in first episode and drug-naïve patients with schizophrenia: Negative correlation with cognitive impairment. *Psychiatry Res*. 2021;304(January 2021). doi:10.1016/j.psychres.2021.114164

26. Anhøj S, Nielsen MØ, Jensen MH, et al. Alterations of intrinsic connectivity networks in

antipsychotic-naïve first-episode schizophrenia. *Schizophr Bull*. 2018;44(6):1332-1340. doi:10.1093/schbul/sbx171

27. Huang ML, Khoh TT, Lu SJ, et al. Relationships between dorsolateral prefrontal cortex metabolic change and cognitive impairment in first-episode neuroleptic-naive schizophrenia patients. *Med (United States)*. 2017;96(25). doi:10.1097/MD.0000000000007228

28. Wei Q, Yan W, Zhang R, Yang X, Xie S. Aberrant cortical surface and cognition function in drug-naive first-episode schizophrenia. *Ann Gen Psychiatry*. 2022;21(1):1-10. doi:10.1186/s12991-022-00381-7

29. Guo X, Li J, Wang J, et al. Hippocampal and orbital inferior frontal gray matter volume abnormalities and cognitive deficit in treatment-naive, first-episode patients with schizophrenia. *Schizophr Res*. 2014;152(2-3):339-343. doi:10.1016/j.schres.2013.12.015

30. Wu JQ, Chen DC, Tan YL, et al. Cognitive impairments in first-episode drug-naive and chronic medicated schizophrenia: MATRICS consensus cognitive battery in a Chinese Han population. *Psychiatry Res*. 2016;238:196-202. doi:10.1016/j.psychres.2016.02.042

31. Guo Q, Hu Y, Zeng B, et al. Parietal memory network and default mode network in first-episode drug-naïve schizophrenia: Associations with auditory hallucination. *Hum Brain Mapp*. 2020;41(8):1973-1984. doi:10.1002/hbm.24923

32. Zhang C, Cai J, Zhang J, et al. Genetic modulation of working memory deficits by ankyrin 3 gene in schizophrenia. *Prog Neuro-Psychopharmacology Biol Psychiatry*. 2014;50:110-115. doi:10.1016/j.pnpbp.2013.12.010

33. Zhuo K, Lu Y, Yang Z, et al. Prospective memory performance in patients with drug-naïve, first-episode psychosis. *Schizophr Res*. 2013;143(2-3):285-290. doi:10.1016/j.schres.2012.12.002

34. Yoon JH, Westphal AJ, Minzenberg MJ, et al. Task-evoked substantia nigra hyperactivity associated with prefrontal hypofunction, prefrontonigral disconnectivity and nigrostriatal connectivity predicting psychosis severity in medication naïve first episode schizophrenia. *Schizophr Res*. 2014;159(2-3):521-526. doi:10.1016/j.schres.2014.09.022

35. Zhao J, Zhang Y, Liu F, Chen J, Zhao J, Guo W. Abnormal global-brain functional connectivity and its relationship with cognitive deficits in drug-naive first-episode adolescent-onset schizophrenia. *Brain Imaging Behav*. 2022;16(3):1303-1313. doi:10.1007/s11682-021-00597-3

36. Hu M, Xia Y, Zong X, et al. Risperidone-induced changes in DNA methylation in peripheral blood from first-episode schizophrenia patients parallel changes in neuroimaging and cognitive phenotypes. *Psychiatry Res*. 2022;317(August):114789. doi:10.1016/j.psychres.2022.114789

37. Tao Q, Miao Y, Li H, et al. Insulin Resistance and Oxidative Stress: In Relation to Cognitive Function and Psychopathology in Drug-Naïve, First-Episode Drug-Free Schizophrenia. *Front Psychiatry*. 2020;11(November):1-7. doi:10.3389/fpsyt.2020.537280

38. Andreasen NC, Rezai K, Alliger R, Ii VWS, Flaum M. Hypofrontality in Neuroleptic-Naive Patients and Patients With Chronic Schizophrenia. *Arch Gen Psychiatry*. 1992;49:943-958.

39. Brickman AM, Buchsbaum MS, Bloom R, et al. Neuropsychological functioning in first-break, never-medicated adolescents with psychosis. *J Nerv Ment Dis*. 2004;192(9):615-622. doi:10.1097/01.nmd.0000138229.29157.3e

© 2024 Lee M et al. *JAMA Psychiatry.*

40. Buchsbaum MS, Haier RJ, Potkin SG, et al. Frontostriatal Disorder of Cerebral Metabolism in Never-Medicated Schizophrenics. *Arch Gen Psychiatry*. 1992;49(12):935-942. doi:10.1001/archpsyc.1992.01820120023005

41. Chan RCK, Chen EYH, Law CW. Specific executive dysfunction in patients with first-episode medication-naïve schizophrenia. *Schizophr Res*. 2006;82(1):51-64. doi:10.1016/j.schres.2005.09.020

42. Fagerlund B, Mackeprang T, Gade A, Hemmingsen R, Glenthøj BY. Effects of Low-Dose Risperidone and Low-Dose Zuclopenthixol on Cognitive Functions in First-Episode Drug-Naive Schizophrenic Patients. *CNS Spectr*. 2004;9(5):364-375.

43. Finkelstein JRJ, Cannon TD, Gur RE, Gur RC, Moberg P. Attentional dysfunctions in neuroleptic-naive and neuroleptic-withdrawn schizophrenic patients and their siblings. *J Abnorm Psychol*. 1997;106(2):203-212. doi:10.1037//0021-843X.106.2.203

44. He Z, Deng W, Li M, et al. Aberrant intrinsic brain activity and cognitive deficit in first-episode treatment-naive patients with schizophrenia. *Psychol Med*. 2013;43(4):769-780. doi:10.1017/S0033291712001638

45. Hill SK, Beers SR, Kmiec JA, Keshavan MS, Sweeney JA. Impairment of verbal memory and learning in antipsychotic-naïve patients with first-episode schizophrenia. *Schizophr Res*. 2004;68(2-3):127-136. doi:10.1016/S0920-9964(03)00125-7

46. Hill SK, Schuepbach D, Herbener ES, Keshavan MS, Sweeney JA. Pretreatment and longitudinal studies of neuropsychological deficits in antipsychotic-naïve patients with schizophrenia. *Schizophr Res*. 2004;68(1):49-63. doi:10.1016/S0920-9964(03)00213-5

47. Hilti CC, Delko T, Orosz AT, et al. Sustained attention and planning deficits but intact attentional set-shifting in neuroleptic-naïve first-episode schizophrenia patients. *Neuropsychobiology*. 2010;61(2):79-86. doi:10.1159/000265133

48. Hong KS, Kim JG, Koh HJ, et al. Effects of risperidone on information processing and attention in first-episode schizophrenia. *Schizophr Res*. 2002;53(1-2):7-16. doi:10.1016/S0920-9964(01)00167-0

49. Hu M, Chen J, Li L, et al. Semantic fluency and executive functions as candidate endophenotypes for the early diagnosis of schizophrenia in Han Chinese. *Neurosci Lett*. 2011;502(3):173-177. doi:10.1016/j.neulet.2011.07.037

50. Krieger S, Lis S, Cetin T, Gallhofer B, Meyer-lindenberg A. Executive Function and Cognitive Subprocesses in First-Episode, Drug-Naive Schizophrenia: An Analysis of N-Back Performance. *Am J Psychiatry*. 2005;(June):1206-1208.

51. Lu W, Zhang C, Yi Z, Li Z, Wu Z, Fang Y. Association between BDNF Val66Met polymorphism and cognitive performance in antipsychotic-naïve patients with schizophrenia. *J Mol Neurosci*. 2012;47(3):505-510. doi:10.1007/s12031-012-9750-4

52. Nejad AB, Ebdrup BH, Siebner HR, et al. Impaired temporoparietal deactivation with working memory load in antipsychotic-naÃve patients with first-episode schizophrenia. *World J Biol Psychiatry*. 2011;12(4):271-281. doi:10.3109/15622975.2010.556199

53. Parellada E, Catarineu S, Catafau A, Bernardo M, Lomeña F. Psychopathology and Wisconsin card

© 2024 Lee M et al. *JAMA Psychiatry.*

sorting test performance in young unmedicated schizophrenic patients. *Psychopathology*. 2000;33(1):14-18. doi:10.1159/000029113

54. Salgado-Pineda P, Baeza I, Pérez-Gómez M, et al. Sustained attention impairment correlates to gray matter decreases in first episode neuroleptic-naive schizophrenic patients. *Neuroimage*. 2003;19(2):365-375. doi:10.1016/S1053-8119(03)00094-6

55. Van Veelen NMJ, Vink M, Ramsey NF, van Buuren M, Hoogendam JM, Kahn RS. Prefrontal lobe dysfunction predicts treatment response in medication-naive first-episode schizophrenia. *Schizophr Res*. 2011;129(2-3):156-162. doi:10.1016/j.schres.2011.03.026

56. Wang Q, Chan R, Sun J, et al. Reaction time of the Continuous Performance Test is an endophenotypic marker for schizophrenia: A study of first-episode neuroleptic-naive schizophrenia, their non-psychotic first-degree relatives and healthy population controls. *Schizophr Res*. 2007;89(1-3):293-298. doi:10.1016/j.schres.2006.08.030

57. Zhang XY, Chen DC, Xiu MH, et al. Gender differences in never-medicated first-episode schizophrenia and medicated chronic schizophrenia patients. *J Clin Psychiatry*. 2012;73(7):1025-1033. doi:10.4088/JCP.11m07422

58. Wang H, Zhang B, Zeng B, et al. Association between catechol-O-methyltransferase genetic variation and functional connectivity in patients with first-episode schizophrenia. *Schizophr Res*. 2018;199:214-220. doi:10.1016/j.schres.2018.04.023

59. Vyas NS, Buchsbaum MS, Lehrer DS, et al. D2/D3 dopamine receptor binding with [F-18]fallypride correlates of executive function in medication-naïve patients with schizophrenia. *Schizophr Res*. 2018. doi:10.1016/j.schres.2017.05.017

© 2024 Lee M et al. *JAMA Psychiatry.*
