# Supplementary material for: Cognitive Function and Variability in Antipsychotic Drug–Naive Patients With First-Episode Psychosis: A Systematic Review and Meta-Analysis
Source: JAMA Psychiatry. 2024 Feb 28;81(5):468–76. doi: 10.1001/jamapsychiatry.2024.0016 (PMC10902783; doi:10.1001/jamapsychiatry.2024.0016)
Supplement: Supplement 2. — Data Sharing Statement. [file jamapsychiatry-e240016-s002.pdf]

Data Sharing Statement

Lee. Cognitive Function and Variability in Antipsychotic Drug–Naive Patients With First-Episode Psychosis. *JAMA Psychiatry*. Published February 28, 2024.
doi:10.1001/jamapsychiatry.2024.0016

Data
Data available: No

Additional Information
Explanation for why data not available: The vast majority of data is already published and openly accessible. Code for reproducing the analyses and figures can be found at https://github.com/MariaLeeR/Meta_analysis_cogn_dnFEP
